# Supporting Informed Vaccine Decision-Making and Communication in Pregnancy Through the Vaccines in Pregnancy Canada Intervention: Multimethod Co-Design Study

**DOI:** 10.2196/77446

**Published:** 2025-12-16

**Authors:** Eliana Castillo, Andrea M Patey, Medea Myers-Stewart, Monica Santosh Surti, Maria Castrellon Pardo, Marcia Bruce, Zaileen Jamal, Madison Kennedy, Julie A Bettinger, Jessica Kaufman, Margie Danchin, Maoliosa Donald

**Affiliations:** 1 Department of Medicine, Cumming School of Medicine University of Calgary Calgary, AB Canada; 2 Department of Obstetrics and Gynaecology, Cumming School of Medicine University of Calgary Calgary, AB Canada; 3 Alberta Children's Hospital Research Institute University of Calgary Calgary, AB Canada; 4 Department of Medicine, Quality and Safety IWK Health Halifax, NS Canada; 5 Vaccine Evaluation Center, BC Children's Hospital Research Institute University of British Columbia Vancouver, BC Canada; 6 Department of Pediatrics, Faculty of Medicine University of British Columbia Vancouver, BC Canada; 7 Department of Paediatrics The University of Melbourne Melbourne Australia; 8 Vaccine Uptake Group Murdoch Children's Research Institute Melbourne, Victoria Australia; 9 Department of General Medicine Royal Children's Hospital Melbourne, Victoria Australia; 10 Department of Community Health Sciences Cumming School of Medicine University of Calgary Calgary, AB Canada

**Keywords:** behavioral sciences, decision-making, digital health, maternal vaccination, patient-centered care, pregnancy, prenatal care, vaccination hesitancy, vaccination, vaccine communication

## Abstract

**Background:**

Vaccination in pregnancy (VIP) protects pregnant individuals and their newborns; yet, uptake remains suboptimal. Pregnant individuals face unique decision-making challenges, and communication with their health care provider (HCP) is crucial for uptake. While there is extensive data on barriers to VIP, interventions applying evidence-based behavior change strategies and co-designed with end users are scarce. Our prior work indicated that a new Canadian intervention was needed.

**Objective:**

This study aimed to co-design a multicomponent intervention to support informed decision-making and vaccine communication in pregnancy.

**Methods:**

Our multimethod study followed the Double Diamond phases (ie, Discover, Define, Develop, and Deliver) and partnered with a diverse patient advisory council and a multidisciplinary team of HCPs. During the Discover and Define phases, our previous work, we explored gaps and barriers to VIP in Canada and defined the behavior change strategies to address those needs. During the Develop phase, we co-designed and conducted iterative prototyping of four intervention components: (1) a pregnancy-specific communication approach, (2) a skills course for HCPs, (3) a practice change plan, and (4) a website with evidence-based resources for patients and HCPs. We used online and in-person participatory co-design sessions and peer-to-peer, patient-oriented online focus groups and semistructured in-depth interviews. During the Deliver phase, we refined the intervention components through functionality and usability testing.

**Results:**

The Vaccines in Pregnancy Canada (VIP Canada) intervention consists of four integrated components: (1) DECIDE (Determine, Elicit, Consent, Interactive discussion, Deliver, and Empower): a patient-centered, pregnancy-specific communication approach for providers to deliver a clear vaccine recommendation while respecting autonomy. (2) Skills course for HCPs: 4 self-paced, online modules to learn the rationale for VIP and the DECIDE communication approach and 2 group sessions. Providers found the skills course clear, practical, and applicable across diverse clinical roles and settings. Feedback led to enhancements, including improved audio-visual synchronization, consistent closed captioning, and the addition of downloadable reference materials to support learning. (3) Practice change plan: an action plan HCPs make to integrate vaccine communication into their practice. (4) VIP Canada website: an evidence-based website with resources to support informed vaccine decision-making for patients and providers. Patient feedback informed iterative refinements to the layout and content of the website to enhance navigation, readability, and representation of diverse identities. Functionality and usability testing demonstrated that patients found the VIP Canada website visually appealing, easy to navigate, and supportive of informed decision-making.

**Conclusions:**

The VIP Canada is a promising intervention co-designed to drive behavior change by addressing key barriers to vaccine communication and informed decision-making around our patient partners’ and HCPs’ perspectives and lived experiences to bridge theoretical frameworks with real-world relevance. Next steps include a feasibility study for further refinement and a subsequent effectiveness study.

## Introduction

Vaccination in pregnancy (VIP) keeps parents and babies safer by preventing infection-related morbidity and mortality [1-4]. Despite these benefits, vaccine uptake in pregnancy remains low in Canada and globally, particularly after the COVID-19 pandemic, which exacerbated vaccine hesitancy and mistrust in health care systems [5,6].

Even though there is extensive data on barriers to VIP [7-14] and VIP decision-making [15,16], there is limited literature regarding interventions that apply evidence-based strategies to address these barriers. A recent systematic review and meta-analysis of VIP interventions for influenza, pertussis, tetanus, and COVID-19 was carried out, including 36 randomized and nonrandomized quasi-experimental studies in 15 high- and low-middle-income countries [17]. The study found very limited evidence on the effect of existing interventions to improve VIP uptake [17]. However, 2 systematic reviews looking at the effectiveness of interventions to improve influenza VIP uptake in high-income countries [18,19], 1 focusing on the effect of digital interventions [19], suggested that educational interventions for pregnant individuals, like pamphlets, websites, and brief one-to-one education, can be effective. Digital interventions can be more effective than nondigital or no intervention at all, and digital interventions should include videos, social media, and e-books, rather than text messages [19]. These reviews highlighted the lack of high-quality studies and the high heterogeneity of existing ones, underscoring the need for further research and better intervention design.

A VIP intervention that has demonstrated positive outcomes is the Sharing Knowledge About Immunization (SKAI), an Australian intervention developed prior to the COVID-19 pandemic by patients and experts to provide credible, evidence-based vaccine information using a presumptive vaccine communication approach [20-23]. A SKAI evaluation study described an increase in self-reported pregnancy and childhood vaccination uptake [21-24], leading to its use in primary care clinics nationally in Australia, with Commonwealth government support. SKAI was developed for the Australian context, and further investigation was needed to identify if this intervention could be adopted as is in Canada. Therefore, we undertook a program of research to understand if the SKAI was appropriate for the Canadian setting and met the needs of Canadian health care providers (HCPs) and patients.

We used the Double Diamond process model [25] and the associated framework for innovation design principles [25] to guide our research program activities. The Double Diamond encompasses four phases: (1) Discover, which refers to problem understanding; (2) Define, which is about summarizing and making sense of the Discover findings toward developing solutions; (3) Develop, which refers to co-designing solutions through iterative co-design cycles; and (4) Deliver, which refers to refining solutions, validation, and usability testing. This paper reports on the last 2 phases, Develop and Deliver; however, we provide a summary (Multimedia Appendix 1) highlighting the findings from multiple studies and activities from the Discover and Define phases [26-31].

Our findings from previous phases indicated that a new intervention tailored specifically for the Canadian context was required, instead of adopting SKAI, for the following reasons: (1) a mismatch between the needs of Canadian patients and HCPs and the components of SKAI, as documented by our behavioral sciences–informed inquiry [26,27,30]; (2) the need for a communication approach that would balance the advantages of both a presumptive communication (as seen in SKAI, where the provider presumes the patient will accept vaccination and provides a leading statement [32]) and participatory communication approaches (ie, where the provider explores the patient’s intentions and supports them in making an informed decision, in the tradition of shared decision-making and motivational interviewing [33]); (3) the need for better digital interventions to improve VIP uptake [19]; and (4) the need to include content pertaining to new vaccines that were not available when SKAI was originally developed (eg, COVID-19 and respiratory syncytial virus).

In the following text, we report on the co-design (Develop phase) and testing (Deliver phase) of a new multicomponent intervention, Vaccines in Pregnancy Canada (VIP Canada), aimed at supporting and improving informed vaccine decision-making and communication during pregnancy. The components that were developed and tested include (1) a novel communication approach, (2) a VIP skills course for HCPs to learn and practice the communication approach, (3) a practice change plan to help HCPs use their skills in practice, and (4) a public-facing website with videos and other digital tools to support patients and providers. A feasibility study is currently underway and will be reported separately.

## Methods

### Study Objectives, Study Design, and Theoretical Frameworks

**Figure 1:**
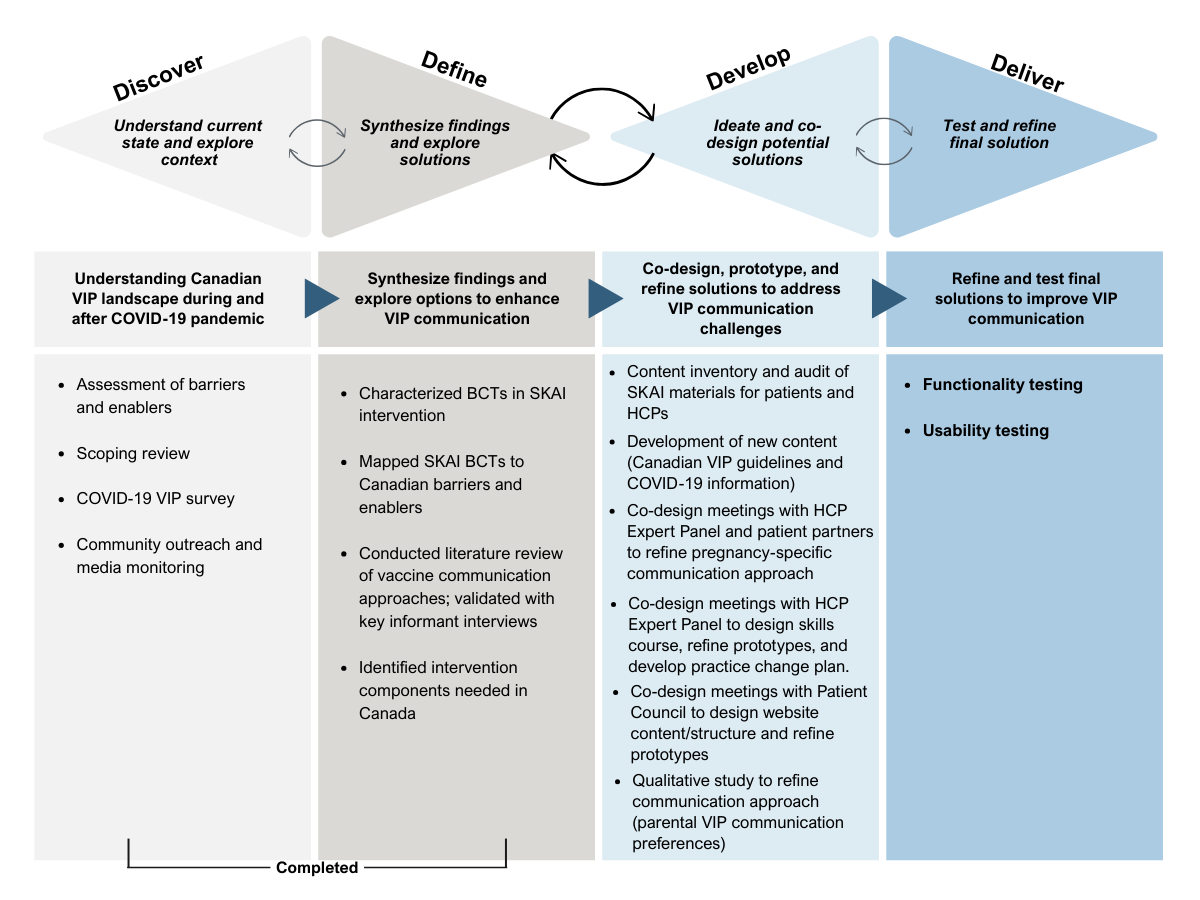
Double Diamond framework activities by phase. Adapted from Design Council (framework for innovation) [34]. BCT: behavior change technique; HCP: health care provider; SKAI: Sharing Knowledge About Immunization; VIP: vaccination in pregnancy.

The objectives of this study were to co-design the VIP Canada intervention components and test the functionality and usability of these individual components using a multimethod approach. Figure 1 [34] presents the Double Diamond process model [20], aligning our research program activities with each of the phases. During the Discover phase, we used a behavioral sciences–informed approach to identify barriers and enablers to VIP communication in Canada [26-29]. During the Define phase we determined if the behavior change strategies required to address the identified Canadian needs were met by the SKAI intervention [30], conducted key informant interviews to confirm the need for a communication approach, and defined the 4 intervention components that would be required to address Canadian needs. In this paper, we report on the Develop and Deliver phases. During the Develop phase, we co-designed the 4 intervention components with end users (parents and providers) through iterative co-design cycles, a qualitative inquiry on parental communication preferences, and content revision and creation. Finally, during the Deliver phase, we tested the functionality and usability of the intervention components.

We followed a rigorous behavioral sciences–informed process for problem understanding because doing so increases the likelihood of intervention effectiveness [35] by identifying barriers and enablers that influence behavior and behavior change and by selecting strategies that theoretically and empirically address the barriers or enablers of the desired behavior [36,37]. We used co-design, a participatory approach, to coproduce solutions incorporating the lived experiences and knowledge of end users and engaging them as active and equal collaborators in the design process [30-32]. Co-design is grounded in power-sharing, inclusivity, capacity-building, reciprocity, and shared ownership [30] and operates on the idea that end users shape practical and contextually relevant solutions for real-world settings [30-33].

### Study Team

Our core study team included 2 patient partners (MCP and MB) trained in Patient and Community Engagement Research (PaCER) [38], 2 implementation scientists (M Donald and AMP), 2 clinicians (EC and M Danchin), 1 knowledge translation specialist (MMS), and 1 equity, diversity, and inclusion specialist (MSS). Study team members facilitated co-design sessions, led data collection and analysis, and supported the iterative development and testing of intervention components.

### Co-Design Partners

Co-design partners contributed their lived experiences as patients and providers to the ideation, prototyping, and iterative refinement of all intervention components. These partners included the following:

1. Resident Patient Council, established in 2021, composed of 10 parents with lived experience of pregnancy, representing diverse populations, gender identities, and cultural backgrounds.
2. Multidisciplinary HCP Expert Panel, established in 2023, composed of 15 perinatal providers, including nurses, pharmacists, physicians, midwives, and doulas.
3. Additional patient and provider partners (20 patient partners and 19 provider partners) who supported specific development and testing activities.

The HCP Expert Panel and the Patient Council are integral parts of our study team, who actively contribute to direction-setting and decision-making across all phases of the research process. Their ongoing involvement reflects our commitment to patient-oriented research principles [39] and ensures our work is person-centered, inclusive, and responsive to real-world needs.

### Participants

Patients and HCPs were recruited to participate in relevant activities within the Develop and Delivery phases. Patient participants met the following inclusion criteria: planning a pregnancy, currently pregnant, recently postpartum, or nursing; aged 18 years or older; and residing in Canada. Recruitment was primarily conducted through social media platforms, such as Facebook and Instagram. Perinatal HCPs were recruited through purposive snowball sampling across 6 Canadian provinces (Alberta, Ontario, New Brunswick, Manitoba, Quebec, and British Columbia) and included nurses, midwives, doulas, pharmacists, physicians, and a lactation consultant.

### Develop Phase: Activities and Methods

#### Overview

The Develop phase focused on operationalizing, prototyping, and refining the intervention components we identified as priorities for the Canadian context during the Define phase, including a patient-facing website, a provider skills course, a pregnancy-specific communication approach, and a provider practice change plan. We conducted a structured review of the SKAI website, created new content, conducted focus groups and interviews with patients regarding parental communication preferences [31], and held 11 iterative co-design sessions with end users (4 with the Patient Council and patient partners and 7 with the multidisciplinary HCP Expert Panel). This work was iterative in nature.

#### SKAI Content Review and Creation of New Content

Study team members (MMS, MSS, and EC) developed a structured Microsoft Excel–based inventory of SKAI’s patient-facing website and accompanying resources for HCPs. The team reviewed the content page by page (website) and resource by resource to identify areas requiring adaptation for the Canadian context, including clinical information such as recommended vaccines, immunization schedules, and available products. Proposed adaptations were recorded in the inventory alongside the rationale for adaptation. We also identified topics where new content would need to be developed to meet the needs of Canadian HCPs and patients, like COVID-19 vaccinations, and subsequently confirmed in co-design sessions with the HCP Expert Panel and Patient Council. Study team members (MMS and MSS) synthesized Canadian national guidelines and drafted new content that was reviewed by the study team’s clinical expert (EC) and HCP partners (nurses, midwives, and pharmacists) and continued to be reviewed and refined based on feedback from the Patient Council and HCP Expert Panel.

#### Co-Design Meetings With the HCP Expert Panel, Patient Council, and Patient Partners

We held a total of 11 co-design meetings (8 online and 3 in-person) to develop and refine the 4 components of the intervention. All co-design meetings were facilitated by study team members (MB, MCP, MSS, and MMS), including 2 patient partners (MB and MCP) trained in PaCER [38], using participatory tools, including commentary charts [40] and virtual whiteboards. Online meetings were 60-90 minutes in length, conducted over Zoom (Zoom Communications, Inc), recorded with consent, transcribed, and supplemented with facilitator notes. In-person sessions were 90 minutes in length. One in-person session included unscripted interactions with patient partners that were filmed with consent and later included in the provider course’s online modules as demonstration videos. After each meeting, the core study team (EC, MB, MCP, MSS, and MMS) synthesized feedback, reached consensus on revisions, and refined prototypes accordingly. We used the Absorb Learning Management System (Absorb Software Inc) to house the providers’ course and Whimsical UX (Whimsical Inc) to build the initial website wireframe, as well as Squarespace (Squarespace, Inc) to develop the functional prototypes.

#### Focus Groups and Interviews on Parental Communication Preferences

On the recommendation of our Patient Council and to support the development of the pregnancy-specific communication approach and the website’s name, tone, format, and presentation, we conducted several focus groups and interviews. These included 4 semistructured focus groups and 4 individual interviews with 14 participants identifying as currently pregnant, recently pregnant, or planning a pregnancy from diverse cultural backgrounds between June and July 2023 [31]. Details on participant characteristics, recruitment, methods, and data analysis are reported in Castrellon Pardo et al [31].

### Deliver Phase: Activities and Methods

The Deliver phase assessed the functionality and usability of the VIP Canada website and the provider skills course to ensure overall alignment with end user needs.

#### Functionality Testing

We conducted functionality testing of the website and the online modules of the provider skills course between December 2023 and May 2024 with the HCP Expert Panel, the Patient Council, and additional patient and HCP partners to identify basic problems with navigation, layout, video, or image quality prior to usability testing. For the website, we conducted a structured survey (Multimedia Appendix 2) consisting of yes or no questions, open-text responses for each website section, and Likert scale questions to assess overall user experience. For the provider skills course, testers were asked to complete each of the online modules using the learning management system’s (LMS) “reviewer” function and provide feedback on basic functionality (Multimedia Appendix 3).

#### Usability Testing

##### Overview

We conducted usability testing between February and July 2024 with research participants, including both patients and HCPs who were not previously involved in co-design or functionality testing, to capture end users’ perspectives on the design, content, and overall usability of the VIP Canada website and provider skills course. Feedback was used to identify and resolve design, content, and usability issues before launching the intervention for the feasibility study, which is currently underway.

##### VIP Canada Website Usability Testing

We conducted one-on-one 60-minute Zoom interviews with patients recruited via community networks and social media (Multimedia Appendix 4) facilitated by 2 study team members (MCP and ZJ), including 1 trained in PaCER. Participants were asked to complete scenario-based tasks (eg, locating trimester-specific vaccine information, identifying support resources) while following a think-aloud protocol [41,42], where they verbalized their thoughts, reactions, and decision-making processes. Interviews were transcribed verbatim and deidentified prior to analysis.

##### VIP Skills Course Usability Testing

HCP research participants recruited purposively through our networks, with support from our expert panel, completed the online course modules individually and responded to (1) module-specific evaluation questionnaires in the LMS and (2) an overall course evaluation questionnaire after completing all the modules (Multimedia Appendix 5). Survey items included Likert-scale and open-text questions to evaluate layout and navigation, content adequacy and comprehensiveness, readability, user experience, and satisfaction, as recommended for e-learning usability and quality evaluation [43-45]. We also asked participants about the inclusivity of language and visuals to reflect priorities identified by our patient and HCP partners during the co-design process. Survey responses were collected electronically through the LMS and deidentified prior to analysis. Lastly, we conducted 2 virtual sessions and one in-person session with HCP partners to further evaluate and refine the role-play scenarios and debriefing tool used in the group practice component of the VIP skills course. Feedback and suggestions for improvement were captured through detailed notes taken by study team members.

##### Functionality and Usability Data Analysis

Quantitative data from the website and course functionality and usability testing were analyzed using descriptive statistics. We used a content analysis approach [46] for the open-text survey responses and the think-aloud interviews. We categorized key issues and prioritized refinements to content, design, and overall functionality of the website and the VIP skills course prior to formal usability testing and repeated the process prior to launching the intervention for the feasibility study. Issues and suggestions for refinement were categorized as either (1) immediate priority changes to be made prior to usability testing and then before launch or (2) future refinements to be addressed later. Our final decisions were based on factors such as the importance of each change to the overall user experience and the estimated time and resources needed to implement them.

### Ethical Considerations

This work was approved by the University of Calgary Conjoint Health Research Ethics Board under ethics certificates REB21-1055, REB21-1465, REB23-1277.

All participants received verbal and written information about the study to facilitate an informed and voluntary decision regarding participation. Informed consent was obtained from each participant, either in physical or electronic form prior to study involvement.

Participant data were kept confidential and securely stored at the local research site. Only the local study team had access to participants’ personal information. Data were deidentified, and participants were assigned unique study numbers that contained no identifying information. Consistent with the participatory nature of some stages, participants were offered the option to be acknowledged in resulting reports or to remain anonymous.

All study members had completed Tri-Council Policy Statement-2 and Alberta Health Services Data and Privacy and Confidentiality training, ensuring awareness of their responsibilities and safeguarding participants’ privacy and confidentiality.

## Results

### Overview

We collaborated with our patient and HCP partners (n=43) and used data collected from research participants (n=42) for a total of 85 patients and providers to co-design and test the digital components of the VIP Canada intervention. All research patient participants (n=19) self-identified as women; 21% (n=4) were pregnant, and 74% (n=14) had recently given birth or were breastfeeding; 79% (n=15) were 28-37 years of age; 79% (n=15) reported living in urban areas; and self-reported ethnocultural backgrounds were 37% (n=7) Caucasian, 32% (n=6) South Asian, 16% (n=3) Latin American, and 16% (n=3) Indigenous, Filipino, or multiracial. All research HCP participants (n=23) self-identified as women; 48% (n=11) were family physicians, and 35% (n=8) were nurses or midwives; 87% (n=20) practiced in urban areas; and 70% (n=16) had been practicing for more than 10 years.

**Figure 2:**
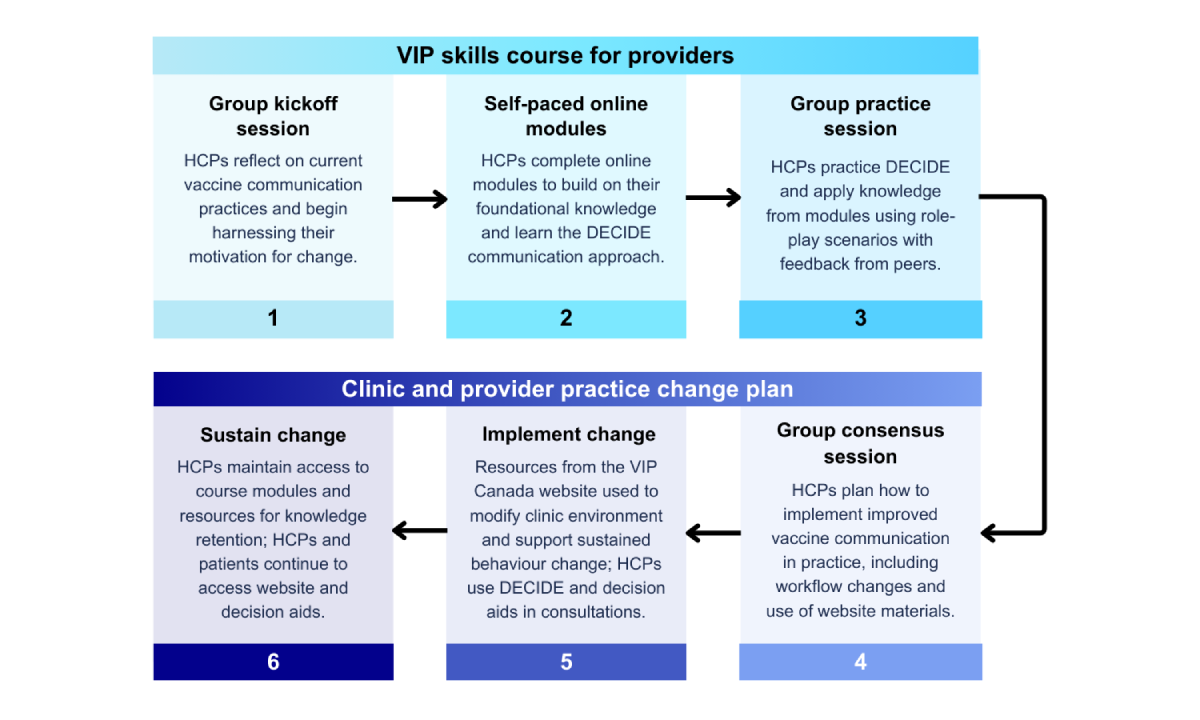
Vaccines in Pregnancy Canada (VIP Canada) intervention implementation. DECIDE: Determine, Elicit, Consent, Interactive discussion, Deliver, and Empower; HCP: health care provider.

The intervention includes the following four interrelated components: (1) the DECIDE (Determine, Elicit, Consent, Interactive discussion, Deliver, and Empower) communication approach, (2) the VIP skills course for HCPs, (3) the practice change plan, and (4) the VIP Canada website. Together, the intervention components aim to guide patients and HCPs toward meaningful behavior change (Figure 2).

### Develop Phase: Results of Prototyping and Refining Intervention Components

#### Pregnancy-Specific Communication Approach (DECIDE)

DECIDE is a patient-centered, pregnancy-specific communication approach for HCPs to deliver a clear vaccine recommendation while respecting patient autonomy based on shared decision-making principles [47]. We have previously reported on how we developed DECIDE [47]. DECIDE combines a participatory communication style [48] with a clear vaccine recommendation to meet patients’ desire for both autonomy and a clear recommendation in decision-making [47], as informed by parental communication preferences focus groups and interviews [31] and co-design sessions with Patient Council and HCP Expert Panel. DECIDE stands for “Determine” if your patient is aware and ready to discuss vaccination, “Elicit” your patient’s questions, “Consent” to share information and engage in a discussion about vaccination, “Interactive discussion” to address your patient’s specific questions, “Deliver” a vaccine recommendation that considers your patient’s perspectives, and “Empower” your patient to take next step toward an informed decision [47]. The HCP Expert Panel confirmed that the final DECIDE approach was more realistic, relevant, and user-friendly than earlier communication approach prototypes that required providers to select either a participatory [48] or a presumptive [48] communication style based on their perception of a patient’s level of hesitancy. HCPs found DECIDE’s shared decision-making approach valuable in the postpandemic context, as vaccine conversations are more challenging and providers feel more hesitant to initiate them for fear of damaging patient relationships [26].

I think post-COVID it’s more important to use this approach versus a prescriptive approach, because the general public has been through a lot of directives and lots of mandatory vaccinations and lots of mandates... I think that the [DECIDE] approach will continue to encourage the public to trust us. It just leaves space, recognizing not everybody is at the same place and really developing a relationship where [patients] can trust you as somebody who can share some information in a nonthreatening way.

HCP Expert Panel member

#### VIP Skills Course for Providers

**Figure 3:**
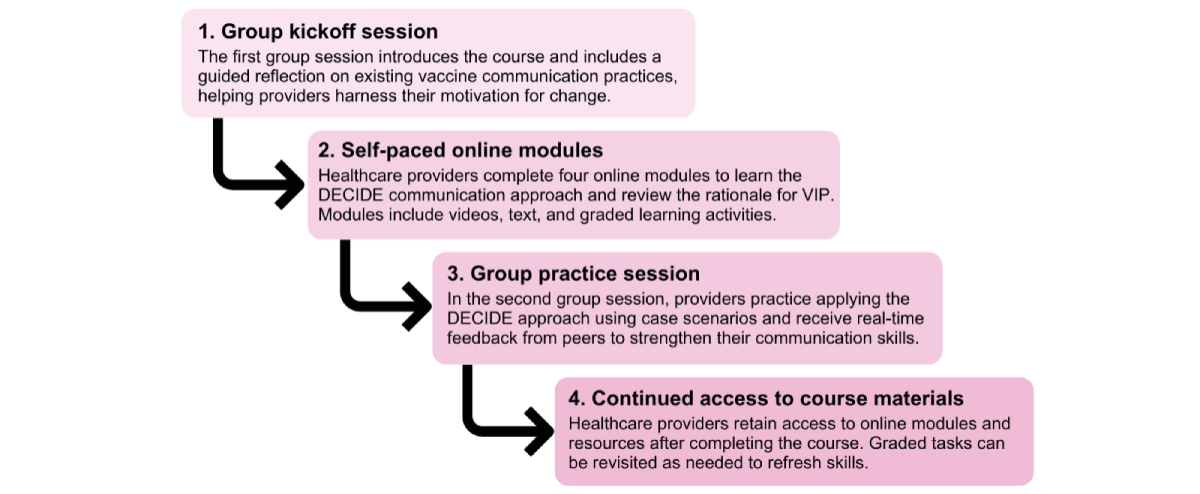
Structure of the vaccination in pregnancy (VIP) skills course for providers. DECIDE: Determine, Elicit, Consent, Interactive discussion, Deliver, and Empower.

The VIP skills course consists of 4 self-paced online modules and 2 group sessions (Figure 3). The first 60-minute kickoff session (delivered online or in person) introduces HCPs to course objectives and involves a guided reflection on their VIP communication practices and experiences, harnessing their motivation to change. This is followed by 4 self-paced online learning modules to learn the rationale for VIP and the DECIDE communication approach and how to apply it. The course concludes with a second group practice session (delivered online or in person) where HCPs practice their communication skills using role-play scenarios and receive feedback from their peers. The 2 group sessions are facilitated by an HCP or other staff member in each clinic using content provided in the LMS to organize the sessions and lead discussions.

Grounded in evidence-based adult learning theory and behavioral sciences, the course equips providers with (1) knowledge (eg, rationale for VIP or Canadian VIP recommendations), (2) the DECIDE communication approach, and (3) how to use it in their everyday practice. The course targets the gaps and barriers to VIP communication identified with Canadian patients and HCPs, using behavior change techniques (BCTs) [36] known to target those barriers, as per the behavioral science–informed inquiry we conducted during the Discover and Define phases [26,27,30,36] (Multimedia Appendix 6). For example, the online modules incorporate quizzes that provide HCPs with immediate feedback (BCT: feedback on behavior) and video demonstrations of the DECIDE approach in practice (BCT: demonstration of behavior); the group practice session gives HCPs the opportunity to apply their skills through role-play (behavioral practice or rehearsal) and peer feedback, all targeting HCPs’ skills, a major barrier identified in the Canadian context [26]. Table 1 provides other examples illustrating how each component of the intervention addresses a barrier and the BCT used to do so.

The structure and delivery format of the course were iteratively co-designed with the HCP Expert Panel (n=15) and informed by the focus groups and interviews on parental VIP communication preferences with patient participants (n=14) [31] and patient partners. Both HCPs and patients emphasized the importance of introducing vaccination early and often during pregnancy to help normalize vaccine conversations and give patients enough time to make decisions. We added these insights to the VIP skills course as key communication tips. HCPs also recommended that the course offer a mix of self-paced and group components to balance flexibility with opportunities for practical skill application and structured feedback following practice activities, which led to the coadaptation of a structured debriefing tool (Multimedia Appendix 7). The final course is an accredited continuing medical education activity by the Royal College of Physicians and Surgeons of Canada and the College of Family Physicians of Canada.

**Table 1:** Vaccines in Pregnancy Canada (VIP Canada) intervention: examples of behavioral targets and techniques. See Multimedia Appendix 6 for a full inventory of descriptions.

VIP Canada intervention component and description				Behavior change techniques^a^ [30,37]		Barrier to VIP^b^ in Canada [26]
**DECIDE communication approach**						
	**DECIDE acronym**					
		The acronym gives clear guidance on the steps that the health care providers need to follow to effectively use the communication approach.	Instructions on how to perform the behavior		Skills Knowledge	
**VIP skills course for providers**						
	**Module 1: Why vaccinate in pregnancy?**					
		Animations that review the rationale for VIP, including suggested readings to support information provided and a call-to-action inviting providers to engage in VIP communication.	Information about antecedents Information about health consequences		Beliefs about consequences Social professional role and identity Knowledge	
**Provider practice change plan**						
	**Consensus session**					
		Providers generate a plan to integrate vaccine communication into their practice and make a commitment to follow it. They identify who will initiate the VIP conversations, when and where, and the environment modifications needed (eg, which materials are needed in the physical space to support the plan).	Goal setting (behavior) Goal setting (outcome) Action planning Commitment		Environmental context and resources Social professional role and identity Intentions	
**VIP Canada website**						
	**FAQ^c^** **, vaccine-specific content, and trimester-specific sections**					
		Contains information on how vaccines work, their components, safety, myths vs facts, and the importance of vaccination during pregnancy. Clearly states recommended timing and what to expect. Features quotes from patients that allow pregnant people to relate with the experiences of their peers.	Information about health consequences and antecedents Social comparison Instructions on how to perform a behavior		Beliefs about consequences Social influences Knowledge	

^a^Behavior change techniques are observable, replicable, and irreducible components of an intervention designed to alter or redirect causal processes that regulate behavior.

^b^VIP: vaccination in pregnancy.

^c^FAQ: frequently asked questions.

#### Provider Practice Change Plan

The practice change plan was co-designed with HCP partners (n=13) to support providers in thinking through how to incorporate vaccine communication into their routine prenatal care and how to execute it effectively. It engages providers in structured action planning, leverages their intentions and commitment to change, and supports environmental modifications by using materials (eg, waiting room posters) and decision aids (eg, infographics, vaccine timelines) from the VIP Canada website as visual cues to enable and sustain behavior change. HCP partners highlighted challenges including workflow integration and team-based coordination. They also emphasized the value of building consensus among clinical teams when determining how workflows and other internal processes should be adapted to support effective vaccine communication. Based on this input, we drafted a structured planning template (Multimedia Appendix 8) to help provider teams map out what changes they will implement and how to do it. By assisting HCPs in determining when, where, and how they will integrate vaccine communication into their routine practice, the practice change plan aims to bridge the gap between intention and practice [49] and supports alignment and consistency within clinics and among providers, which also helps to reinforce behavior change.

#### VIP Canada Website

The VIP Canada website is an evidence-based digital resource that supports informed vaccine decision-making for pregnant individuals, their families, and HCPs looking for easy-to-access resources to support vaccine communication and decision-making in real time. The VIP Canada website is listed as a trusted resource by national and provincial health organizations in Canada, including Immunize Canada [50] and HealthLink BC [51]. The website integrates elements adapted from the SKAI intervention along with newly codeveloped content tailored to the Canadian context. It addresses the emotional and social influences on vaccine decision-making identified by Canadian patients and HCPs in our previous research [26,30] (Multimedia Appendix 6). The website was iteratively refined through co-design sessions with Patient Council members (n=10) and informed by focus groups and interviews we held with patients on their VIP communication preferences (n=14) [31], focusing on the following five key areas: (1) layout and organization: patients recommended presenting vaccine information by trimester, so we created a “Vaccines by Trimester” landing page and added “Key Facts” sections to each page to help users quickly locate relevant information. We also adapted 4 vaccine-specific infographics from the SKAI intervention and refined them with the Patient Council to present key information in a visual format. (2) Content: patients identified content gaps related to vaccinations before and after pregnancy and the importance of support from social networks when making vaccine decisions, so we developed pages on recommended vaccinations before pregnancy, after delivery, and during breastfeeding and a “Support Person” page with resources and tips for partners, families, and friends. (3) Tone and messaging: patients highlighted the importance of using supportive language and messaging that emphasizes autonomy and reinforces the protective benefits of vaccination for both the pregnant person and their baby. This was used to guide the site’s overall tone and contributed to the codevelopment of the slogan, “Protection for you is protection for two.” (4) Diversity and representation: patients emphasized the value of seeing themselves and their diverse lived experiences reflected in the visuals, graphics, and videos on the website. This led us to replace generic stock photos with images depicting more diverse families, cultural backgrounds, gender identities, and body types. (5) Website name and branding: participants preferred the term “vaccines” over “immunizations” to enhance accessibility for individuals who speak English as an additional language. They also favored the acronym “VIP” (Vaccines in Pregnancy), which they described as both catchy and affirming to pregnant individuals. Based on this input, we selected “Vaccines in Pregnancy Canada” and the abbreviated “VIP Canada” as the website name and public-facing identity of the intervention.

### Deliver Phase: Results of Functionality and Usability Testing

#### VIP Skills Course

The VIP skills course and the embedded DECIDE communication approach were evaluated through (1) functionality and usability testing of the 4 online course modules to assess technical performance and user experience and (2) guided role-play sessions to test and refine the group practice session (Figure 3) where HCPs practice their communication skills and receive peer feedback.

#### VIP Skills Course Online Modules Functionality Results

Functionality testing of the online course modules was conducted before broader usability testing by study team members (MB, MCP, and ZJ), HCP Expert Panel members (n=6), and additional provider partners (n=5). We made revisions to audio clips and closed captioning, and decreased excessive manual clicking. We added downloadable outlines and reference lists to each of the online modules, as testers found it helpful to have reference materials they could download and use while navigating through the course.

#### VIP Skills Course Online Modules Usability Results

We conducted usability testing of the online course modules with 10 HCP research participants, including nurses (n=4), midwives (n=2), a pharmacist (n=1), a doula or lactation consultant (n=1), a family physician specializing in low-risk obstetrics care (n=1), and an adult learning specialist (n=1). All 10 participants completed the module-specific evaluations, and 9 completed the overall course evaluation survey (Multimedia Appendix 5).

Findings from the overall course evaluation survey indicated high participant satisfaction with the layout and navigability of the course (Table 2). All respondents (n=9, 100%) reported that the course was either “easy” (n=3, 33%) or “very easy” (n=6, 67%) to navigate, with a mean rating of 4.67 (SD 0.50). Similarly, all participants rated the overall course layout positively as either “good” (n=1, 11%) or “excellent” (n=8, 89%), with a mean rating of 4.89 (SD 0.33).

In terms of course content, qualitative feedback from HCP participants described the content as relevant for a diverse range of providers and practice settings.

The content and descriptions are thorough and to the point. The [DECIDE] abbreviations and steps for vaccination in pregnancy [discussion] were exciting and promising.

HCP participant

Participants also appreciated how the course struck a balance between clinical detail and accessible language and found the reflective questions and key messages helpful for remembering core concepts.

I really like how the information is introduced in this module. There is a reason, followed by the rationale. The language is clear and well-balanced in terms of “medicalese,” given the target audience.

HCP participant

Regarding areas for improvement, survey responses documented mixed views on the depth of the content. While most participants agreed the content was in-depth enough to understand the subject matter (agree: n=2, 22%; strongly agree: n=5, 56%), 2 (22%) participants disagreed, resulting in a mean of 4.11 (SD 1.27). Participants recommended adding more in-depth content on vaccine timing, coadministration, and the physiology of maternal antibody transfer.

I would love a more in-depth discussion of the physiology of the maternal antibodies and how this is mother nature’s way of protection. I understood but I felt like I was looking for a deeper understanding.

HCP participant

Participants also recommended incorporating more ethnocultural diversity into the images and other visuals to enhance inclusivity and suggested removing overly gendered visuals (eg, female physicians wearing pink scrubs).

We used these insights to guide several modifications to the final course, such as adding content on vaccine timing, coadministration, and the mechanisms of maternal antibody transfer. We also revised multiple images and graphic illustrations, including patient and provider personas, to reflect greater ethnocultural and gender diversity.

**Table 2:** Usability testing: overall course satisfaction survey ratings (n=9). Percentages are calculated row-wise.

Survey item			Ratings										
			1, n (%)		2, n (%)		3, n (%)		4, n (%)		5, n (%)		Mean (SD)
**Agreement scale (1=strongly disagree, 2=disagree, 3=neutral, 4=agree, 5=strongly agree)**													
	Menus or options were intuitive	0 (0)		0 (0)		0 (0)		6 (67)		3 (33)		4.33 (0.50)	
	Amount of information was manageable	0 (0)		0 (0)		0 (0)		8 (89)		1 (11)		4.11 (0.33)	
	Amount of information was adequate	0 (0)		0 (0)		1 (11)		6 (67)		2 (22)		4.11 (0.60)	
	Covered all expected topics	0 (0)		1 (11)		0 (0)		5 (56)		3 (33)		4.11 (0.93)	
	Content was in-depth enough	0 (0)		2 (22)		0 (0)		2 (22)		5 (56)		4.11 (1.27)	
	Language was inclusive	0 (0)		0 (0)		2 (22)		3 (33)		4 (44)		4.22 (0.83)	
	Images were inclusive	0 (0)		0 (0)		2 (22)		3 (33)		4 (44)		4.22 (0.83)	
**Quality scale (1=very poor, 2=poor, 3=neutral, 4=good, 5=excellent)**													
	Overall course layout	0 (0)		0 (0)		0 (0)		1 (11)		8 (89)		4.89 (0.33)	
	Readability of text or content	0 (0)		0 (0)		0 (0)		3 (33)		6 (67)		4.67 (0.50)	
**Difficulty scale (1=very difficult, 2=difficult, 3=neutral, 4=easy, 5=very easy)**													
	Course navigation	0 (0)		0 (0)		0 (0)		3 (33)		6 (67)		4.67 (0.50)	
**Satisfaction scale (1=very dissatisfied, 2= dissatisfied, 3=neutral, 4=satisfied, 5=very satisfied)**													
	Satisfaction with online platform	0 (0)		0 (0)		0 (0)		4 (44)		5 (56)		4.56 (0.53)	

#### Guided Role-Play Sessions to Test the Group Practice Component of the VIP Skills Course

##### Overview

Results of 2 virtual role-play sessions and 1 in-person role-play session with provider partners (n=7) and study team members (MCP, MB, and MSS) confirmed the relevance and usability of the group practice session format, role-play scenarios, and debriefing tool. Providers described the role-play scenarios as realistic and aligned with their real-world interactions with pregnant patients. We used the feedback from these sessions to inform key refinements, such as adding the DECIDE communication steps to the scenario cards for providers to have on hand while role-playing. We also introduced additional conversation endings to help providers wrap up conversations effectively and incorporated alternative scenario outcomes to reflect diverse patient responses. Providers found the debriefing tool (Multimedia Appendix 7) useful for guiding group reflection and discussion after the role-play exercises. Following iterative updates after each session, we confirmed that the format of the group practice session was appropriate and feasible for both in-person and virtual delivery.

##### Provider Practice Change Plan

Drafts of the implementation roadmap, practice change template, and guidelines for conducting the HCP consensus session were reviewed by the study team, 1 HCP partner and 13 HCP research participants, to confirm they were clear, relevant, and acceptable. The usability of the practice change plan is being tested as part of an ongoing feasibility study.

##### VIP Canada Website Functionality Testing Results

A total of 11 patient partners (out of 17 who started) completed functionality testing by reviewing each page of the VIP Canada website on Squarespace and using a structured survey (Multimedia Appendix 2) with yes or no questions, followed by open-text responses for each section and Likert scale questions for the website in general. Patient partners highly rated the website’s ease of navigation, intuitiveness, aesthetics, and page loading. There was disagreement on the amount of content, suggesting the need to replace text with graphic alternatives (Table 3). They praised the look and feel of the website and described the “Vaccines by Trimester” navigation option as helpful for finding information that was specific to their individual needs and stage of pregnancy.

**Table 3:** Website functionality testing ratings (n=11). Ratings were on a 5-point Likert scale (1=strongly disagree to 5=strongly agree). Percentages are calculated row-wise.

Survey item	Strongly disagree, n (%)	Somewhat disagree, n (%)	Neither agree nor disagree, n (%)	Somewhat agree, n (%)	Strongly agree, n (%)	Mean (SD)
Easy to navigate	0 (0)	0 (0)	0 (0)	2 (18)	9 (82)	4.82 (0.40)
Website is intuitive	0 (0)	0 (0)	0 (0)	4 (36)	7 (64)	4.64 (0.50)
Pages loaded quickly (n=10)^a^	0 (0)	0 (0)	0 (0)	2 (20)	8 (80)	4.80 (0.42)
Amount of content was appropriate	0 (0)	1 (9)	2 (18)	3 (27)	5 (46)	4.09 (1.04)
Pages are aesthetically pleasing	0 (0)	0 (0)	0 (0)	3 (27)	8 (73)	4.73 (0.47)

^a^One participant did not provide a rating for “Pages loaded quickly,” resulting in n=10 for that item; all other items had n=11.

##### VIP Canada Website Usability Testing Results

A total of 6 patient research participants completed usability testing following the think-aloud protocol [41,42]. They described the website as clear and visually appealing, liked the inclusive language, representative imagery, and nonjudgmental tone, and agreed that key messages (eg, “Answering your questions and giving you evidence-based information to make your decisions about vaccination during pregnancy”) were supportive of individual choice and autonomy without being overly prescriptive. Table 4 reports on difficulty ratings for specific tasks. Although zero frustration was the most frequent rating across all tasks, some participants had a harder time with tasks that asked them to find information using specific keywords, such as “mRNA” and “COVID-19” due to the absence of a keyword search function, which our team was still developing at the time of testing. No tasks reached a 3-failure rating.

Participants suggested improvements such as integrating a dedicated search bar and updating the “Browse Topics” headings to make them clearer and easier to navigate. They also found the parent testimonial videos and quotes to be very relatable and suggested adding more to reflect a more diverse range of lived experiences and ethnocultural backgrounds. Lastly, participants recommended adding more videos and graphics to break up sections with dense text to improve overall readability. Based on this feedback, we integrated a site-wide search function using Google’s Programmable Search Engine, which allows users to search website content by keyword. We engaged our Patient Council to codevelop additional patient decision aids, including a downloadable VIP timeline and an infographic on respiratory syncytial virus, to help break down key information in a visually appealing format. We also added more parent testimonials (videos and quotes) across the website to reflect a broader range of lived experiences and ethnocultural and gender identities.

**Table 4:** Website usability task difficulty ratings (n=6). Participants were tasked to locate specific information on the website. Total ratings reflect the pooled number of ratings (n=30) within each response option across all tasks. Percentages are calculated row-wise.

Task	0 (zero frustration), n (%)	1 (little frustration), n (%)	2 (medium or high frustration), n (%)	3 (point of failure), n (%)	Mean (SD)
Influenza vaccines	5 (83)	1 (17)	0 (0)	0 (0)	0.17 (0.41)
Vaccines in second trimester	5 (83)	0 (0)	1 (17)	0 (0)	0.33 (0.82)
Support person tips	5 (83)	1 (17)	0 (0)	0 (0)	0.17 (0.41)
COVID-19 vaccines	3 (50)	3 (50)	0 (0)	0 (0)	0.50 (0.55)
mRNA^a^ vaccines	4 (67)	1 (17)	1 (17)	0 (0)	0.50 (0.84)
Total ratings (n=30)	22 (73)	6 (20)	2 (7)	0 (0)	0.33 (0.61)

^a^mRNA: messenger RNA.

## Discussion

### Principal Findings

VIP Canada is an evidence-based, multicomponent intervention designed to support vaccine communication and improve informed decision-making during pregnancy in Canada. VIP Canada was designed to drive behavior change by targeting the barriers to VIP communication and decision-making identified by Canadian patients and HCPs using fit-for-purpose, evidence-based behavior change strategies and practical tools. The final VIP Canada intervention consists of four interrelated components: (1) the DECIDE communication approach, (2) the VIP skills course for HCPs, (3) a practice change plan to support clinic-wide implementation, and (4) a public-facing VIP Canada website with evidence-based information, resources, and patient decision aids. VIP Canada addresses 2 significant gaps in improving VIP acceptance through (1) a pregnancy-specific vaccine communication approach responsive to the postpandemic context and (2) advancing the digital health integration in patient-centered care by developing a skills-based course for HCPs within an LMS platform and a digital platform with videos, animations, and testimonials for patients, providing them with multiple ways to learn skills and access trusted information using evidence-based behavior change techniques, all in one intervention.

We report on the development of VIP Canada through rigorous co-design, grounded in a behavioral and implementation sciences–informed inquiry, and on the functionality and usability testing of its components before the launch of a feasibility study, which is now underway. Usability testing results suggest that VIP Canada is a promising intervention that prioritizes skills development and addresses additional determinants of behavior change, such as implementation intentions and environmental modifications, beyond traditional information–focused websites and educational materials.

Existing interventions, such as the Brief Motivational Interviewing for Maternal Immunizations (MI4MI) [52,53] and SKAI [21,22], have demonstrated promising approaches to addressing gaps in effective VIP communication. The MI4MI intervention aims to improve how providers talk with pregnant people about vaccines using a video and 2 interactive modules. MI4MI guides HCPs to begin with a vaccine recommendation and then use motivational interviewing techniques for patients who express hesitancy. Perinatal clinicians found MI4MI useful and relevant; however, its effectiveness in improving vaccine acceptance or uptake during pregnancy is not known [52]. SKAI, as described in the introduction, reported an increase in self-reported VIP uptake from 43% to 81% [21-24]. VIP Canada builds on the foundation of the SKAI intervention and includes newly co-designed components to meet the needs of HCPs and patients in the Canadian context [30].

In contrast to M14MI’s focus on HCP recommendations, VIP Canada targets behavior change at the levels of the patient, provider, and health care environment because it is also necessary to address patient- and environment-related factors that influence vaccine decision-making during pregnancy (Multimedia Appendix 6).

In contrast to SKAI, VIP Canada promotes a shared decision-making approach to vaccine communication that directly addresses the needs of Canadian HCPs and patients in the postpandemic context, while SKAI recommends starting vaccine conversations with a strong recommendation to position vaccination as the norm and routine standard of care before moving on to address patient questions [54]. VIP Canada’s DECIDE approach recognizes that making decisions about vaccination during pregnancy is complex and that it is normal for pregnant individuals to have questions or concerns about getting vaccinated [47]. DECIDE supports shared decision-making by helping HCPs to deliver clear vaccine recommendations while also empowering patients to take an active role in the process and make choices that reflect their own values and circumstances. Misinformation and declining public confidence in vaccines following the COVID-19 pandemic [55,56] have made it even more essential for HCPs to communicate clearly and respectfully about vaccination without compromising patient trust, which is what the VIP Canada DECIDE approach does.

Ongoing work will advance VIP Canada’s generalizability and inclusivity as necessary steps to inform future spread and scale. First, through our feasibility study examining the acceptability, demand, integration, and fidelity of the VIP Canada intervention in 3 different sites, we are understanding the barriers to implementing VIP Canada in resource-limited and rural settings. Overall, 2 of the 3 feasibility study sites serve rural and remote communities in 2 separate Canadian provinces, including 1 with very low levels of vaccine acceptance. Second, we have established partnerships and convened planning meetings to adapt VIP Canada to the needs of ethnocultural communities with diverse language and cultural requirements. This task was outside the scope of the work presented and requires a different approach and the creation of sustainable partnerships.

### Limitations

While the VIP Canada intervention demonstrates promise, we acknowledge the following limitations: (1) course implementation requirements: the VIP skills course includes 2 group sessions that can be delivered online or in person and require facilitation by an HCP or staff member from each clinic, using content provided by our team. As such, running these sessions successfully depends on a clinic’s capacity and the willingness and comfort of HCPs or other staff to facilitate them, posing additional challenges for clinics that are understaffed or underresourced. We are developing a staff facilitation and training guide to support the consistent delivery of group session material and maintain fidelity across sites. In the future, we may explore alternatives to the group sessions, such as artificial intelligence–simulated online practice modules, to give providers more flexibility and reduce the need for staff facilitation. Furthermore, our feasibility study is looking at implementation outcomes in resource-limited settings and will provide an opportunity to make further adjustments prior to an effectiveness study and spread and scale. (2) Sampling and recruitment: we note that our participants, particularly pregnant people, may not represent diverse groups such as Indigenous individuals and newcomers to Canada; however, ongoing work addresses these gaps. While the urban-rural ratio of our patient partners and research participants is representative of Canada’s, where 80% of the population lives in urban areas, the sample size for usability testing was relatively small, which limits the generalizability of the findings and may mean that we did not capture the full range of perspectives relevant to vaccine communication and decision-making in pregnancy. As patient participants were mainly recruited through social media platforms, individuals with limited access to technology or those with lower digital literacy may have been excluded. Additionally, we recruited HCP participants through purposive snowball sampling, which can introduce bias and limit representativeness by overrepresenting HCPs with similar professional backgrounds, networks, and practice settings [57]. As a mitigation strategy, initial participants were selected from diverse geographical, cultural, and gender backgrounds. Referral chains were monitored to avoid overrepresentation, and purposive recruitment was used where necessary to include underrepresented groups. We acknowledge the limitations of snowballing; however, it continues to be a recommended technique to access hard-to-reach populations. (3) Cultural, linguistic, and practice relevance: while we co-designed the intervention with diverse patient and provider partners, the adaptability of intervention materials across different clinical and cultural contexts may be limited. For example, role-play scenarios may require further tailoring to improve their relevance for practice settings serving Indigenous communities or those in rural and remote areas, and we recognize that the delivery mode of current intervention components is not necessarily equitable (eg, online, in English only, etc). We aim to develop a digital repository of tested and validated role-play scenarios that participants and facilitators can choose from that align better with their clinical context and practice realities. Additionally, the VIP skills course for providers and the patient decision aids are currently only available in English, while the VIP Canada website is offered in English and French. This can be a barrier for speakers of other languages.

### Conclusions

VIP Canada is a promising multicomponent and co-designed intervention designed to address the complexity of vaccine decision-making during pregnancy. Grounded in behavioral and implementation science and developed through iterative engagement with Canadian HCPs and patients, VIP Canada offers practical, tailored solutions for pregnant individuals, their families, and the providers who support them by equipping HCPs with the skills and strategies they need to engage in respectful and patient-centered vaccine communication and empowering patients with accessible, trustworthy resources that support informed decision-making. A feasibility study is underway to examine the acceptability, demand, integration, and fidelity of VIP Canada implementation in 3 sites and partner with patients, intermediaries (eg, multicultural health brokers), and HCPs to co-design adaptations suited to newcomers.

We are continuing to strengthen VIP Canada by partnering with IMPRINT UK (Immunising Pregnant Women and Infants Network) [58]. We are adding animated videos on the benefits and safety of maternal vaccination and testimonials from patients from diverse ethnocultural communities and in other languages, including French, Hindi, Punjabi, Spanish, and Vietnamese. We are also working with partners, including the Multicultural Health Brokers Cooperative [59], to ensure that intervention materials meet the cultural and linguistic needs of all Canadians. Future refinements undertaken by our team may include exploring the potential of artificial intelligence–based practice simulations or other tools to make the VIP skills course more flexible for busy HCP teams.

Future research is needed to evaluate the effectiveness of VIP Canada in improving vaccine uptake and its long-term impact on maternal and fetal health outcomes. Additionally, future studies should explore how the VIP Canada intervention can be applied or adapted to support informed decision-making around other complex topics during pregnancy, such as cannabis or other substance use.

## Appendix

Multimedia Appendix 1 Discover and Define phases.

Multimedia Appendix 2 Website usability testing.

Multimedia Appendix 3 Online course modules functionality testing instructions.

Multimedia Appendix 4 Website usability testing interview guide.

Multimedia Appendix 5 Online course modules usability testing.

Multimedia Appendix 6 VIP Canada intervention behavioral targets and techniques.

Multimedia Appendix 7 Practice session debriefing tool.

Multimedia Appendix 8 Practice change action plan.

## Abbreviations

BCT: behavior change technique
DECIDE: Determine, Elicit, Consent, Interactive discussion, Deliver, and Empower
HCP: health care provider
IMPRINT: Immunising Pregnant Women and Infants Network
LMS: learning management system
MI4MI: Brief Motivational Interviewing for Maternal Immunizations
PaCER: Patient and Community Engagement Research
SKAI: Sharing Knowledge About Immunization
VIP Canada: Vaccines in Pregnancy Canada
VIP: vaccination in pregnancy

## Data Availability

These data are the product of a multiple encounters, including peer-to-peer research conducted by patient research partners. The datasets generated or analyzed during this study are available from the corresponding author on reasonable request. Note, this work falls under more than one ethics certificate, some of which do not allow for the sharing of data. Requests for access to deidentified and aggregated data supporting our conclusions can be made to the corresponding author and will be considered on a case-by-case basis in accordance with ethical guidelines and participants consent form.
