# Supplementary material for: Supporting Informed Vaccine Decision-Making and Communication in Pregnancy Through the Vaccines in Pregnancy Canada Intervention: Multimethod Co-Design Study
Source: J Med Internet Res. 2025 Dec 16;27:e77446. doi: 10.2196/77446 (PMC12754583; doi:10.2196/77446)
Supplement: Multimedia Appendix 1 [file jmir_v27i1e77446_app1.pdf]

Appendix 1 _ **Discover and Define Phases – Activities and Key Findings to Inform Intervention Design**

Activity	Sample Size	Overview & Methods	Key Findings That Informed Intervention Design
Scoping Review[1]	n/a	Scoping review of resources available for patient-provider vaccine communication in Canada following JBI methodology.[2, 3]	Most VIP resources available to HCPs in Canada focus on providing information rather than building communication skills.
Barrier and Enabler Assessment[4]	N=169 patient participants<br>N= 47 providers	Qualitative study of barriers and enablers to vaccine communication during pregnancy in Canada using the Theoretical Domains Framework.[5]	Canadian HCPs lack confidence and skills in VIP communication and worry about damaging their relationships with patients. Patients struggle with concerns about VIP perceived safety, amount of information and discerning what is best for them and their babies.
Survey on Psychological antecedents to COVID-19 VIP Decisions [6]	N=237 patient participants from Alberta.	Quantitative study using the 5C scale for the psychological antecedents to vaccine decision-making[7] and self-reported COVID-19 VIP.	COVID-19 vaccination during pregnancy was strongly associated with Confidence, Complacency and Collective Responsibility, receiving a non-COVID-19 vaccination during pregnancy, and trust in healthcare providers.
Identifying Behavioral Intervention Components Needed to address VIP gaps in Canada[8]	n/a	Multi-method study using behavioural tools [9, 10][11] to identify Behaviour Change Techniques in an existing intervention [12], map them to VIP barriers and enablers in Canada and identify additional ones that could improve VIP in Canada.	Existing intervention primarily targeted barriers related knowledge deficits rather than *Skills and Beliefs about capabilities* for Canadian HCPs and *Emotions, Social influences and Reinforcement* for patients.
Qualitative enquiry on COVID-19 vaccination in ethnocultural communities [13, 14]	N= 28 intermediaries (cultural health brokers)	Participatory action mixed-methods study using SenseMaker (Cognitive Edge) platform [15, 16] to explore the COVID-19 vaccination response in ethnocultural communities	Trust, safe spaces for collective sensemaking, relationships and cultural and social capital work to reduce decisional conflict and misinformation in ethnocultural communities.
Community Outreach to identify VIP needs	20+ events	Community engagement through town halls, webinars, community fairs (Arabic, South Asian, Ethiopian, Eritrean and Filipino communities), and social media monitoring of parents' groups	Parents valued peers lived experiences and narratives. They want VIP shared decision-making supported by trusted HCPs, intermediaries, and social networks, and resources that reflect their lived experiences.
Literature Review and Key Informant Interviews	4 experts on vaccine communication approaches	Literature review of existing vaccine communication approaches and validation of literature review findings with key informants.	Both presumptive and participatory approaches offer advantages, but neither fully meets parent–provider needs.

1. Surti, M.S., et al., *Resources available for parent-provider vaccine communication in pregnancy in Canada: a scoping review.* BMJ Open, 2023. **13**(8): p. e072473.

2. *The Joanna Briggs Institute Reviewers' Manual: 2015 edition*. 2015, The Joanna Briggs Institute.
3. Peters, M.D.J., et al., *Updated methodological guidance for the conduct of scoping reviews.* JBI Evidence Synthesis, 2020. **18**(10): p. 2119-2126.
4. Patey, A.M., et al., *Factors that influence vaccination communication during pregnancy: provider and patient perspectives using the theoretical domains framework.* JBI Evid Implement, 2024.
5. Cane, J., D. O'Connor, and S. Michie, *Validation of the theoretical domains framework for use in behaviour change and implementation research.* Implementation Science, 2012. **7**(1): p. 37.
6. Lee, K., et al., *Understanding COVID-19 vaccination decisions during pregnancy and while breastfeeding in a Canadian province.* Expert review of vaccines, 2023. **22**(1): p. 520-527.
7. Betsch, C., et al., *Beyond confidence: Development of a measure assessing the 5C psychological antecedents of vaccination.* PLoS One, 2018. **13**(12): p. e0208601.
8. Patey, A.M., et al., *Using behaviour change techniques to identify a short list of intervention components for adapting of vaccination in pregnancy communication intervention in Canada.* JBI Evidence Implementation (Forthcoming), 2025.
9. Project, T.H.B.C. *Theory & Techniques Tool*. 2020 [cited 2021 March]; Available from: https://theoryandtechniquetool.humanbehaviourchange.org.
10. Michie, S., L. Atkins, and R. West, *The Behaviour Change Wheel: A Guide To Designing Interventions*. First ed. 2014, United Kingdom: Silverback Publishing.
11. Michie, S., et al., *The behavior change technique taxonomy (v1) of 93 hierarchically clustered techniques: building an international consensus for the reporting of behavior change interventions.* Ann Behav Med, 2013. **46**(1): p. 81-95.
12. Kaufman, J., et al., *Designing a multi-component intervention (P3-MumBubVax) to promote vaccination in antenatal care in Australia.* Health Promot J Austr, 2020. **32**: p. 391-396.
13. Castillo, E., et al., *Reducing decisional conflict in COVID-19 vaccination in ethnocultural communities through sensemaking: a participatory action mixed-methods study.*, in *Canadian Immunization Conference*. November 27, 2024: Ottawa, ON.
14. Campbell-Scherer, D., et al. *Empowering trusted intermediaries to navigate the complex challenges of COVID-19 vaccination in ethnocultural communities.* in *52nd North American Primary Care Research Group - NAPCRG Annual Meeting*. 2024. Québec City, Canada.
15. Rancati A, S.D., *Managing complexity (and chaos) in times of crisis. A field guide for decision makers inspired by the Cynefin framework [Internet]*, ed. P.O.o.t.E. Union. 2021, Luxembourg.
16. Snowden D, G.Z., Blignaut S., *Cynefin - weaving sense-making into the fabric of our world.*, ed. B.B. Greenberg R. 2021, Singapore, United States, United Kingdom.: Cognitive Edge - The Cynefin Co. 376.
