# Supplementary material for: Supporting Informed Vaccine Decision-Making and Communication in Pregnancy Through the Vaccines in Pregnancy Canada Intervention: Multimethod Co-Design Study
Source: J Med Internet Res. 2025 Dec 16;27:e77446. doi: 10.2196/77446 (PMC12754583; doi:10.2196/77446)
Supplement: Multimedia Appendix 2 [file jmir_v27i1e77446_app2.pdf]

UNIVERSITY OF CALGARY

Default Question Block

Welcome to the VIP CANADA Heuristics and functionality assessment. Please answer this questionnaire while you navigate the website: https://www.vaccinesinpregnancycanada.ca/ (enter "pemba" as the password to log in). Thank you in advance for your collaboration.

Navigation details

Where are you accessing the VIP Canada website from?

- ○ Mobile phone
- ○ Laptop
- ○ Desktop
- ○ Tablet

Which browser are you using?

- ○ Google Chrome
- ○ Apple Safari
- ○ Microsoft Edge
- ○ Mozilla Firefox
- ○ Opera
- ○ Internet Explorer
- ○ Other

ABOUT

Click to write the question text

- ○ Yes
- ○ No

Please describe what didn't work as expected in the "ABOUT" section or any additional feedback related to it.

Are there any typos in the "ABOUT" section?

- Yes
- No

Please describe any typos you found in the "ABOUT" section (please try to be as specific as you can, e.g. specify the heading, question, or paragraph and the word to be corrected).

Do the content and images in the "ABOUT" section fit your screen without being cut off or distorted?

- Yes
- No

Please describe which content or images in the "ABOUT" section do not fit your screen or any additional feedback related to it.

Are the images in the "ABOUT" section clear (e.g. non-pixelated, high-resolution etc.)?

- ○ Yes
- ○ No

Please describe which images on the section on the section "ABOUT" are not clear or any additional feedback related to it.

Do you feel that the images and content in the "ABOUT" section are appropriate (e.g. respectful, inclusive and relevant)?

- ○ Yes
- ○ No

Please specify which images or content in the "ABOUT" section you feel are not appropriate and explain why or add any additional feedback related to it

Do you feel the reading level in the "ABOUT" section is suitable for the intended audience (pregnant people and support persons of pregnant people across Canada)?

- ◯ Yes
- ◯ No

Please specify which content in the "ABOUT" section is unsuitable for the intended audience (i.e. pregnant people and support persons of pregnant people across Canada) and why or any additional feedback related to it.

HOMEPAGE

Please navigate to the HOMEPAGE by clicking on the VIP Canada Logo on the top left of the webpage and answer the following questions:

When I click interactive content (e.g. clickable links) on the HOMEPAGE, it takes me to the intended destination as described or expected.

- ○ Yes
- ○ No

Please describe what didn't work as expected on the HOMEPAGE or any additional feedback related to it.

Are there any typos on the HOMEPAGE?

- ○ Yes
- ○ No

Please describe any typos you found on the HOMEPAGE, please try to be as specific as you can (e.g. specify the heading, question or paragraph and the word to be corrected).

Do the content and images on the HOMEPAGE fit your screen without being cut off or distorted?

- Yes
- No

Please describe which content or images on the HOMEPAGE do not fit on your screen or any additional feedback related to it.

Are the images on the HOMEPAGE clear (e.g. non-pixelated, high-resolution etc.)?

- ○ Yes
- ○ No

Please describe which images on the HOMEPAGE are not clear or any additional feedback related to it.

Do you feel that the images and content on the HOMEPAGE are appropriate (e.g. respectful, inclusive and relevant)?

- ○ Yes
- ○ No

Please describe which images or content on the HOMEPAGE you feel are inappropriate and explain why or add any additional feedback related to it.

Do you feel the reading level of the HOMEPAGE is suitable for the intended audience (i.e. pregnant people and support persons of pregnant people across Canada)?

- ○ Yes
- ○ No

Please describe which content on the HOMEPAGE is unsuitable for the intended audience (i.e. pregnant people and support persons of pregnant people across Canada) and why or any additional feedback related to it.

GENERAL FAQ

Please navigate to the "GENERAL FAQ" section by clicking on the "I have vaccine questions" tab. Then, answer the following questions:

When I click interactive content (e.g. clickable links) on the

"GENERAL FAQ" section, it takes me to the intended destination as described or expected.

- Yes
- No

Please describe what didn't work as expected on the "GENERAL FAQ" section or any additional feedback related to it.

Are there any typos on the "GENERAL FAQ" section?

- Yes
- No

Please describe any typos you found on the "GENERAL FAQ" section, please try to be as specific as you can (e.g. specify the heading, question, or paragraph and the word to be corrected)

Do the content and images on the "GENERAL FAQ" section fit your screen without being cut off or distorted?

- ○ Yes
- ○ No

Please describe which content or images on the "GENERAL FAQ" section doesn't fit your screen or any additional feedback related to it.

Are the images on the "GENERAL FAQ" section clear (e.g. non-pixelated, high-resolution etc.)?

- ○ Yes
- ○ No

Please describe which images on the section "GENERAL FAQ" are not clear or any additional feedback related to it.

Do you feel that the images and content on the "GENERAL FAQ" section are appropriate (e.g. respectful, inclusive and relevant)?

- Yes
- No

Please describe which images or content on the "GENERAL FAQ" section you feel are not appropriate and explain why or add any additional feedback related to it.

Do you feel the reading level of the section "GENERAL FAQ" is suitable for the intended audience (i.e. pregnant people and support persons of pregnant people across Canada)?

○ Yes
○ No

Please describe which content you feel in the "GENERAL FAQ" section is of an unsuitable reading level for the intended audience (i.e. pregnant people and support persons of pregnant people across Canada) or any additional feedback related to it.

INFLUENZA VACCINE

Please navigate to the "INFLUENZA VACCINE" section by clicking on the "I have vaccine questions" tab. Then, answer the following questions:

When I click interactive content (e.g. clickable links) on the "INFLUENZA VACCINE" section, it takes me to the intended destination as described or expected.

○ Yes
○ No

Please describe what didn't work as expected on the "INFLUENZA VACCINE" section or any additional feedback related to it.

Are there any typos on the "INFLUENZA VACCINE" section ?

- ◯ Yes
- ◯ No

Please describe any typos you found on the "INFLUENZA VACCINE" section please try to be as specific as you can (e.g. specify the heading, question, or paragraph and the word to be corrected)

Does the content and images on the "INFLUENZA VACCINE" section fit on my screen without being cut off or distorted?

- Yes
- No

Please describe why the content or images on the"INFLUENZA VACCINE" section doesn't fit on your screen or any additional feedback related to it.

Are the images on the "INFLUENZA VACCINE" section clear (e.g. non-pixelated, high-resolution etc.)?

- Yes
- No

Please describe which images on the "INFLUENZA VACCINE" section are not clear or any additional feedback related to

it.

Do you feel that the images and content on the "INFLUENZA VACCINE" section are appropriate (e.g. respectful, inclusive, relevant)?

- Yes
- No

Please describe which images or content on the "INFLUENZA VACCINE" section you feel are not appropriate and explain why or add any additional feedback related to it

Do you feel the reading level of the "INFLUENZA VACCINE" section is suitable for the intended audience (i.e. pregnant people and support persons of pregnant people across Canada)?

- Yes
- No

Please describe which content in the "INFLUENZA VACCINE" is of an unsuitable reading level for the intended audience (i.e. pregnant people and support persons of pregnant people across Canada) or any additional feedback related to it.

COVID-19 VACCINE

Please navigate to the section "COVID-19 VACCINE" by clicking on the "I Have Vaccine Questions" tab. Then, answer the following questions:

When I click interactive content (e.g. clickable links) on the "COVID-19 VACCINE" section, it takes me to the intended destination as described or expected.

- Yes

○ No

Please describe what didn't work as expected on the "COVID-19 VACCINE" section or any additional feedback related to it.

Are there any typos on the "COVID-19 VACCINE" section ?

○ Yes
○ No

Please describe any typos you found on the "COVID-19 VACCINE" section, please try to be as specific as you can (e.g. specify the heading, question, or paragraph and the word)

Do the content and images on the "COVID-19 VACCINE" section fit your screen without being cut off or distorted?

- ○ Yes
- ○ No

Please describe which content or images on the "COVID-19 VACCINE" section doesn't fit your screen or any additional feedback related to it.

Are the images on the "COVID-19 VACCINE" section clear (e.g. non-pixelated, high-resolution etc.)?

- ○ Yes
- ○ No

Please describe which images on the "COVID-19 VACCINE" section are not clear or any additional feedback

related to it.

Do you feel that the images and content on the "COVID-19 VACCINE" section are appropriate (e.g. respectful, inclusive, relevant)?

- Yes
- No

Please describe which images or content on the "COVID-19 VACCINE" you feel are not appropriate and explain why or add any additional feedback related to it

Do you feel the reading level of the "COVID-19 VACCINE" section is suitable for the intended audience (i.e. pregnant people and support persons of pregnant people across Canada)?

○ Yes

○ No

Please describe which content on the "COVID-19 VACCINE" section is of an unsuitable reading level for the intended audience (i.e. pregnant people and support persons of pregnant people across Canada) or any additional feedback related to it.

TDAP VACCINE

Please navigate to the "TDAP VACCINE" section by clicking on the "I have vaccine questions" tab. Then, answer the following questions:

When I click interactive content (for example links) on the "TDAP VACCINE" section, it takes me to the intended destination as described or expected.

○ Yes

○ No

Please describe what didn't work as expected on the "TDAP VACCINE" section or any additional feedback related to it.

Are there any typos on the "TDAP VACCINE" section?

○ Yes
○ No

Please describe any typos you found on the "TDAP VACCINE" (please try to be as specific as you can, for example, specify the heading, question, or paragraph and the word)

Does the content and images on the "TDAP VACCINE" section fit on my screen without being cut off or distorted?

- [ ] Yes
- [ ] No

Please describe why the content or images on the "TDAP VACCINE" section doesn't fit on your screen or any additional feedback related to it.

Are the images on the "TDAP VACCINE" section clear?

- [ ] Yes
- [ ] No

Please describe which images on the "TDAP VACCINE" section are not clear or any additional feedback related to it.

Do you feel that the images and content on the "TDAP VACCINE" section are appropriate (e.g. respectful, inclusive and relevant)?

- ◯ Yes
- ◯ No

Please describe which images or content on the "TDAP VACCINE" section you feel are not appropriate and explain why or add any additional feedback related to it

Do you feel the reading level of the "TDAP VACCINE" section is suitable for the intended audience (i.e. pregnant people and support persons of pregnant people across Canada)?

- ◯ Yes
- ◯ No

Please describe why you feel the reading level of the "TDAP VACCINE" section is unsuitable for the intended audience (i.e. pregnant people and support persons of pregnant people across Canada) or any additional feedback related to it.

TRAVEL & OTHER VACCINES

Please navigate to the "TRAVEL & OTHER VACCINES" section by clicking on the "I Have Vaccine Questions" tab. Then, answer the following questions:

When I click interactive content (for example links) on the "TRAVEL & OTHER VACCINES" section, it takes me to the intended destination as described or expected.

- ○ Yes
- ○ No

Please describe what didn't work as expected on the "TRAVEL & OTHER VACCINES" section or any additional feedback related to it.

Are there any typos on the "TRAVEL & OTHER VACCINES" section?

- Yes
- No

Please describe any typos you found on the "TRAVEL & OTHER VACCINES" please try to be as specific as you can (e.g. specify the heading, question, or paragraph and the word)

Do the content and images on the "TRAVEL & OTHER VACCINES" section fit your screen without being cut off or distorted?

- ○ Yes
- ○ No

Please describe why the content or images on the "TRAVEL & OTHER VACCINES" section doesn't fit on your screen or any additional feedback related to it.

Are the images on the "TRAVEL & OTHER VACCINES" section clear?

- ○ Yes
- ○ No

Please describe which images on the "TRAVEL & OTHER VACCINES" section are not clear or any additional feedback

related to it.

Do you feel that the images and content on the "TRAVEL & OTHER VACCINES" section are appropriate (e.g. respectful, inclusive and relevant)?

- Yes
- No

Please describe which images or content on the "TRAVEL & OTHER VACCINES" section you feel are not appropriate and explain why or add any additional feedback related to it

Do you feel the reading level of the section "TRAVEL & OTHER VACCINES" is suitable for the intended audience (i.e. pregnant people and support persons of pregnant people across Canada)?

- ○ Yes
- ○ No

Please describe which content in the "TRAVEL & OTHER VACCINES" section is of an unsuitable reading level for the intended audience (i.e. pregnant people and support persons of pregnant people across Canada) or any additional feedback related to it.

[ ]

BEFORE PREGNANCY

Please navigate to the section "BEFORE PREGNANCY" by clicking on the "I am Considering Vaccination" tab. Then, answer the following questions:

When I click interactive content (e.g. clickable links) on the "BEFORE PREGNANCY" section, it takes me to the intended destination as described or expected.

- ○ Yes

○ No

Please describe what didn't work as expected on the "BEFORE PREGNANCY" section or any additional feedback related to it.

Are there any typos on the "BEFORE PREGNANCY" section?

○ Yes
○ No

Please describe any typos you found on the "BEFORE PREGNANCY" section please try to be as specific as you can e.g. specify the heading, question, or paragraph and the word)

Do the content and images on the section "BEFORE PREGNANCY" fit your screen without being cut off or distorted?

- ○ Yes
- ○ No

Please describe which content or images on the "BEFORE PREGNANCY" section do not fit on your screen or any additional feedback related to it.

Are the images on the "BEFORE PREGNANCY" section clear?

- ○ Yes
- ○ No

Please describe which images on the section on the "BEFORE PREGNANCY" section are not clear or any additional feedback related to it.

Do you feel that the images and content on the "BEFORE PREGNANCY" section are appropriate (respectful, inclusive, relevant)?

- Yes
- No

Please describe which images or content on the "BEFORE PREGNANCY" section you feel are not appropriate and explain why or add any additional feedback related to it

Do you feel the reading level of the "BEFORE PREGNANCY" section is suitable for the intended audience (i.e. pregnant people and support persons of pregnant people across Canada)?

- Yes
- No

Please describe which content you feel "BEFORE PREGNANCY" section is of an unsuitable reading level for the intended audience (i.e. pregnant people and support persons of pregnant people across Canada) or any additional feedback related to it.

FIRST TRIMESTER

Please navigate to the "FIRST TRIMESTER" section by clicking on the "I am Considering Vaccination" tab. Then, answer the following questions:

When I click interactive content (e.g. clickable links) on the section "FIRST TRIMESTER", it takes me to the intended destination as described or expected.

- ○ Yes
- ○ No

Please describe what didn't work as expected on the "FIRST TRIMESTER" section or any additional feedback related to it.

Are there any typos on the "FIRST TRIMESTER" section?

- ○ Yes
- ○ No

Please describe any typos you found on the "FIRST TRIMESTER" section (please try to be as specific as you can, for example, specify the heading, question, or paragraph and the word)

Does the content and images on the "FIRST TRIMESTER" section fit on my screen without being cut off or distorted?

- ○ Yes
- ○ No

Please describe why the content or images on the "FIRST TRIMESTER" section doesn't fit on your screen or any additional feedback related to it.

Are the images on the "FIRST TRIMESTER" section clear?

- ○ Yes
- ○ No

Please describe which images on the section on the "FIRST TRIMESTER" section are not clear or any additional feedback related to it.

Do you feel that the images and content on the "FIRST TRIMESTER" section are appropriate (respectful, inclusive, relevant)?

- ○ Yes
- ○ No

Please describe which images or content on the "FIRST TRIMESTER" section you feel are not appropriate and explain why or add any additional feedback related to it

Do you feel the reading level of the "FIRST TRIMESTER" section is suitable for the intended audience (i.e. pregnant people and support persons of pregnant people across Canada)?

- ○ Yes
- ○ No

Please describe why you feel the reading level of the "FIRST TRIMESTER" section is unsuitable for the intended

audience (i.e. pregnant people and support persons of pregnant people across Canada) or any additional feedback related to it.

SECOND TRIMESTER

Please navigate to the "SECOND TRIMESTER" section by clicking on the "I am Considering Vaccination" tab. Then, answer the following questions:

When I click interactive content (for example links) on the "SECOND TRIMESTER" section, it takes me to the intended destination as described or expected.

- ○ Yes
- ○ No

Please describe what didn't work as expected on the "SECOND TRIMESTER" section or any additional feedback related to it.

Are there any typos on the "SECOND TRIMESTER" section?

- ○ Yes
- ○ No

Please describe any typos you found on the "SECOND TRIMESTER" section please try to be as specific as you can, e.g. specify the heading, question, or paragraph and the word.

Does the content and images on the "SECOND TRIMESTER" section fit on my screen without being cut off or distorted?

- ○ Yes
- ○ No

Please describe why the content or images on the "SECOND TRIMESTER" section doesn't fit on your screen or any additional feedback related to it.

Are the images on the "SECOND TRIMESTER" section clear?

- Yes
- No

Please describe which images on the "SECOND TRIMESTER" section are not clear or any additional feedback related to it.

Do you feel that the images and content on the "SECOND TRIMESTER" section are appropriate (e.g. respectful, inclusive and relevant)?

- Yes

○ No

Please describe which images or content on the "SECOND TRIMESTER" section you feel are not appropriate and explain why or add any additional feedback related to it

Do you feel the reading level of the "SECOND TRIMESTER" section is suitable for the intended audience (i.e. pregnant people and support persons of pregnant people across Canada)?

○ Yes
○ No

Please describe why you feel the reading level of the "SECOND TRIMESTER" section is unsuitable for the intended audience (i.e. pregnant people and support persons of pregnant people across Canada) or any additional feedback related to it.

THIRD TRIMESTER

Please navigate to the "AFTER DELIVERY & BREASTFEEDING" section by clicking on the "I am Considering Vaccination" tab. Then, answer the following questions:

When I click interactive content (for example links) on the "AFTER DELIVERY & BREASTFEEDING" section, it takes me to the intended destination as described or expected.

- ○ Yes
- ○ No

Please describe what didn't work as expected on the "AFTER DELIVERY & BREASTFEEDING" section or any additional feedback related to it.

Are there any typos on the "AFTER DELIVERY & BREASTFEEDING" section?

- Yes
- No

Please describe any typos you found on the "AFTER DELIVERY & BREASTFEEDING" section please try to be as specific as you can e.g. specify the heading, question, or paragraph and the word

Do the content and images on the "AFTER DELIVERY & BREASTFEEDING" section fit on my screen without being cut off or distorted?

- Yes
- No

Please describe which content or images on the "AFTER DELIVERY & BREASTFEEDING" do not fit your screen or any

additional feedback related to it.

Are the images on the "AFTER DELIVERY & BREASTFEEDING" section clear?

- Yes
- No

Please describe which images on the "AFTER DELIVERY & BREASTFEEDING" section are not clear or any additional feedback related to it.

Do you feel that the images and content on the "AFTER DELIVERY & BREASTFEEDING" section are appropriate (respectful, inclusive, relevant)?

- Yes
- No

Please describe which images or content on the "AFTER DELIVERY & BREASTFEEDING" section you feel are not appropriate and explain why or add any additional feedback related to it.

Do you feel the reading level of the section "AFTER DELIVERY & BREASTFEEDING" is suitable for the intended audience (i.e. pregnant people and support persons of pregnant people across Canada)?

- ◯ Yes
- ◯ No

Please describe which content on the section "AFTER DELIVERY & BREASTFEEDING" section is unsuitable for the intended audience (i.e. pregnant people and support persons of pregnant people across Canada) or any additional feedback related to it.

I AM A SUPPORT PERSON

Please navigate to the section "I AM A SUPPORT PERSON" by clicking on the corresponding tab. Then, answer the following questions:

When I click interactive content (for example links) on the "I AM A SUPPORT PERSON" section, it takes me to the intended destination as described or expected.

- Yes
- No

Please describe what didn't work as expected on the "I AM A SUPPORT PERSON" section or any additional feedback related to it.

Are there any typos on the "I AM A SUPPORT PERSON" section ?

- ○ Yes
- ○ No

Please describe any typos you found on the "I AM A SUPPORT PERSON" section please try to be as specific as you can (e.g. specify the heading, question, or paragraph and the word)

Does the content and images on the "I AM A SUPPORT PERSON" section fit on your screen without being cut off or distorted?

- ○ Yes
- ○ No

Please describe which content or images on the "I AM A SUPPORT PERSON" doesn't fit on your screen or any

additional feedback related to it.

Are the images on the "I AM A SUPPORT PERSON" section clear?

- ○ Yes
- ○ No

Please describe which images on the section on the "I AM A SUPPORT PERSON" section are not clear or any additional feedback related to it.

Do you feel that the images and content on the "I AM A SUPPORT PERSON" section are appropriate (respectful, inclusive, relevant)?

- ○ Yes
- ○ No

Please describe which images or content on the "I AM A SUPPORT PERSON" section you feel are not appropriate and explain why or add any additional feedback related to it

Do you feel the reading level of the "I AM A SUPPORT PERSON" section is suitable for the intended audience (i.e. pregnant people and support persons of pregnant people across Canada)?

- ○ Yes
- ○ No

Please describe which content in the "I AM A SUPPORT PERSON" section is of an unsuitable reading level for the intended audience (i.e. pregnant people and support persons of pregnant people across Canada) or any additional feedback related to it.

INFOGRAPHIC: How Do Flu and Tdap Vaccines Work?

Now, you will proceed to assess the resources that are available on the website. For this part, you will need to evaluate each infographic and the corresponding introduction for each of them. Please navigate through the infographics section by clicking on the "Resources" tab located in the top, horizontal navigation menu.

Once there, go to the infographic "How Do Flu and Tdap Vaccines Work?" and answer the following questions.

When I click on the "Download PDF" button, the infographic "How Do Flu and Tdap Vaccines Work?", is downloaded as described or expected.

- ○ Yes
- ○ No

Please describe what didn't work as expected when downloading the infographic "How Do Flu and Tdap

Vaccines Work?" or any additional feedback related to it.

Are there any typos in the infographic "How Do Flu and Tdap Vaccines Work?"?

- ○ Yes
- ○ No

Please describe any typos you found on the infographic "How Do Flu and Tdap Vaccines Work?", please try to be as specific as you can (e.g. specify the heading, question, or paragraph and the word to be corrected)

Do the content and images on the infographic "How Do Flu and Tdap Vaccines Work?" fit on your screen without being cut off or distorted?

- ○ Yes

○ No

Please describe which content or images on the infographic "How Do Flu and Tdap Vaccines Work?" do not fit your screen or any additional feedback related to it.

Are the images on the infographic "How Do Flu and Tdap Vaccines Work?" clear (e.g. non-pixelated or high-resolution)?

○ Yes
○ No

Please describe which images on the infographic "How Do Flu and Tdap Vaccines Work?" are not clear or any additional feedback related to it.

Do you feel that the images and content on the infographic "How Do Flu and Tdap Vaccines Work?" are appropriate (e.g. respectful, inclusive and relevant)?

- ◯ Yes
- ◯ No

Please describe which images or content on the infographic "How Do Flu and Tdap Vaccines Work?" you feel are not appropriate and explain why or add any additional feedback related to it

Do you feel the reading level of the infographic "How Do Flu and Tdap Vaccines Work?" is suitable for the intended audience (i.e. pregnant people and support persons of pregnant people across Canada)?

- ◯ Yes
- ◯ No

Please describe which content in the infographic "How Do Flu and Tdap Vaccines Work?" is of an unsuitable reading level for the intended audience (i.e. pregnant people and support persons of pregnant people across Canada) or any additional feedback related to it.

INFOGRAPHIC: How Do mRNA COVID-19 Vaccines Work?

Please navigate through the infographics section by clicking on the "Resources" tab.
Once there, go to the infographic "How Do mRNA COVID-19 Vaccines Work?" and answer the following questions.

When I click on the "Download PDF" button, the infographic "How Do mRNA COVID-19 Vaccines Work?", is downloaded as described or expected.

- ○ Yes
- ○ No

Please describe what didn't work as expected when downloading the infographic "How Do mRNA COVID-19 Vaccines Work?" or any additional feedback related to it.

[ ]

Are there any typos on the infographic "How Do mRNA COVID-19 Vaccines Work?"?

- ○ Yes
- ○ No

Please describe any typos you found on the infographic "How Do mRNA COVID-19 Vaccines Work?", please try to be as specific as you can (e.g. specify the heading, question, or paragraph and the word to be corrected)

[ ]

Do the content and images on the infographic "How Do mRNA COVID-19 Vaccines Work?" fit your screen without

being cut off or distorted?

- ○ Yes
- ○ No

Please describe which content or images on the infographic "How Do mRNA COVID-19 Vaccines Work?" don't fit on your screen or any additional feedback related to it.

Are the images on the infographic "How Do mRNA COVID-19 Vaccines Work?" clear?

- ○ Yes
- ○ No

Please describe which images on the infographic "How Do mRNA COVID-19 Vaccines Work?" are not clear or any additional feedback related to it.

Do you feel that the images and content on the infographic "How Do mRNA COVID-19 Vaccines Work?" are appropriate (e.g. respectful, inclusive and relevant)?

- Yes
- No

Please describe which images or content on the infographic "How Do mRNA COVID-19 Vaccines Work?" you feel are not appropriate and explain why or add any additional feedback related to it

Do you feel the reading level of the infographic "How Do mRNA COVID-19 Vaccines Work?" is suitable for the intended audience (i.e. pregnant people and support persons of pregnant people across Canada)?

- Yes

○ No

Please describe which content on the infographic "How Do Flu and Tdap Vaccines Work?" is of an unsuitable reading level for the intended audience (i.e. pregnant people and support persons of pregnant people across Canada) or any additional feedback related to it.

INFOGRAPHIC: Risks and Benefits of the Flu Vaccine

Please navigate to the infographics section by clicking on the "Resources" tab. Once there, go to the infographic "Risks and Benefits of the Flu Vaccine" and answer the following questions.

When I click on the "Download PDF" button, the infographic "Risks and Benefits of the Flu Vaccine", is downloaded as described or expected.

○ Yes
○ No

Please describe what didn't work as expected when downloading the infographic "Risks and Benefits of the Flu Vaccine" or any additional feedback related to it.

Are there any typos on the infographic "Risks and Benefits of the Flu Vaccine"

- ◯ Yes
- ◯ No

Please describe any typos you found on the infographic "Risks and Benefits of the Flu Vaccine", please try to be as specific as you can (e.g. specify the heading, question, or paragraph and the word to be corrected.)

Do the content and images on the infographic "Risks and Benefits of the Flu Vaccine" fit your screen without being cut off or distorted?

- ○ Yes
- ○ No

Please describe which content or images on the infographic "Risks and Benefits of the Flu Vaccine" do not fit your screen or any additional feedback related to it.

Are the images on the infographic "Risks and Benefits of the Flu Vaccine" clear?

- ○ Yes
- ○ No

Please describe which images on the infographic "Risks and Benefits of the Flu Vaccine" are not clear or any additional

feedback related to it.

Do you feel that the images and content on the infographic "Risks and Benefits of the Flu Vaccine" are appropriate (e.g. respectful, inclusive and relevant)?

- Yes
- No

Please describe which images or content on the infographic "Risks and Benefits of the Flu Vaccine" you feel are not appropriate and explain why or add any additional feedback related to it.

Do you feel the reading level of the infographic "Risks and Benefits of the Flu Vaccine" is suitable for the intended

audience (i.e. pregnant people and support persons of pregnant people across Canada)?

- ◯ Yes
- ◯ No

Please describe which content on the infographic "Risks and Benefits of the Flu Vaccine" is of an unsuitable reading level for the intended audience (i.e. pregnant people and support persons of pregnant people across Canada) or any additional feedback related to it.

INFOGRAPHIC: Risks and Benefits of the Tdap Vaccine

Please navigate through the infographics section by clicking on the "Resources" tab. Once there, go to the infographic "Risks and Benefits of the Tdap Vaccine" and answer the following questions.

When I click on the "Download PDF" button, the infographic

"Risks and Benefits of the Tdap Vaccine", is downloaded as described or expected.

- ◯ Yes
- ◯ No

Please describe what didn't work as expected when downloading the infographic "Risks and Benefits of the Tdap Vaccine" or any additional feedback related to it.

Are there any typos on the infographic "Risks and Benefits of the Tdap Vaccine"

- ◯ Yes
- ◯ No

Please describe any typos you found on the infographic "Risks and Benefits of the Tdap Vaccine", please try to be as specific as you can (e.g. specify the heading, question, or paragraph and the word to be corrected)

Do the content and images on the infographic "Risks and Benefits of the Tdap Vaccine" fit on your screen without being cut off or distorted?

- ○ Yes
- ○ No

Please describe which content or images on the infographic "Risks and Benefits of the Tdap Vaccine" do not fit your screen or any additional feedback related to it.

Are the images on the infographic "Risks and Benefits of the Tdap Vaccine" clear?

- ○ Yes
- ○ No

Please describe which images on the infographic "Risks and Benefits of the Tdap Vaccine" are not clear or any additional feedback related to it.

Do you feel that the images and content on the infographic "Risks and Benefits of the Tdap Vaccine" are appropriate (e.g. respectful, inclusive and relevant)?

- Yes
- No

Please describe which images or content on the infographic "Risks and Benefits of the Tdap Vaccine" you feel are not appropriate and explain why or add any additional feedback related to it

Do you feel the reading level of the infographic "Risks and Benefits of the Tdap Vaccine" is suitable for the intended audience (i.e. pregnant people and support persons of pregnant people across Canada)?

- ○ Yes
- ○ No

Please describe which content on the infographic "Risks and Benefits of the Tdap Vaccine" is of an unsuitable reading level for the intended audience (i.e. pregnant people and support persons of pregnant people across Canada) or any additional feedback related to it.

GENERAL QUESTIONS

To finalize, please answer these questions about your overall experience:

	Strongly agree	Somewhat agree	Neither agree nor disagree	Somewhat disagree	Strongly disagree
It was easy to navigate	◯	◯	◯	◯	◯
It was intuitive	◯	◯	◯	◯	◯
The pages loaded quickly	◯	◯	◯	◯	◯
The amount of content was appropriate	◯	◯	◯	◯	◯
The pages are aesthetically pleasing	◯	◯	◯	◯	◯

Please share any additional comments about the website:

Powered by Qualtrics
