# Supplementary material for: Supporting Informed Vaccine Decision-Making and Communication in Pregnancy Through the Vaccines in Pregnancy Canada Intervention: Multimethod Co-Design Study
Source: J Med Internet Res. 2025 Dec 16;27:e77446. doi: 10.2196/77446 (PMC12754583; doi:10.2196/77446)
Supplement: Multimedia Appendix 3 [file jmir_v27i1e77446_app3.pdf]

Heuristics and Functionality Testing Instructions

We would like to thank you for contributing to the Heuristics and Functionality Testing of the Vaccine in Pregnancy Communication Skills Course for providers. The purpose of this activity is to ensure the course is working as it have been designed and capture your feedback about it.

To access the course please go to https://vipcanada.myabsorb.ca/#/login and enter the username and password that we have provided.
Once you have logged in, you will see a little envelope icon in the top right corner. Your most recent message includes instructions on how to view the modules as a reviewer. Feel free to make any comments on the slides- you should see that option on the right side where it says 'add comment'.

For each module capture your feedback regarding:

- What didn't work as expected
- Content inconsistencies
- If you find typos or any graphic or image is pixelated or low resolution.
- If something is not suitable for the intended audience and why.
- If the language and tone is understandable, respectful and inclusive.
- Positive insights

If you want to view the course as a learner, you need to go to 'My Courses' on your dashboard to get that experience. If you have any questions, please contact [redacted] at [redacted]

Thanks.

VIP CANADA TEAM
