# Supplementary material for: Supporting Informed Vaccine Decision-Making and Communication in Pregnancy Through the Vaccines in Pregnancy Canada Intervention: Multimethod Co-Design Study
Source: J Med Internet Res. 2025 Dec 16;27:e77446. doi: 10.2196/77446 (PMC12754583; doi:10.2196/77446)
Supplement: Multimedia Appendix 4 [file jmir_v27i1e77446_app4.pdf]

QUESTION GUIDE USABILITY TESTING

(VIP Canada Website Usability Testing)

Pre-Interview introduction and socio-demographic survey

(to be completed before recording begins)

Welcome and thank you for taking the time to complete this website usability testing with us. My name is ___________________ from the University of Calgary. We would like to record this session to help with note taking, but before we start recording, I would like to ask you to complete an anonymous demographic survey. We want to ensure that our intervention considers the needs of all individuals, regardless of their age, gender, race, or ethnic background. We would like to ask you some questions about yourself so that we can better understand your background, is that ok? If yes, the link to it is pasted in the chat box, I will give you some minutes to complete it

Thank you, we will now start the usability testing. I would like to record this part of the session to help with note taking, are you okay if I start recording now?

START RECORDING

This interview should take 60 – 90 minutes. Before we begin, I would just like to confirm your consent to participate in this interview to conduct website usability testing and to have the interview recorded or written out by a scribe?

Just a reminder that your participation is completely voluntary, which means you can end the interview at any time.

The purpose of our session will be to gauge the usability and ease of navigation of the **Canadian Vaccination in Pregnancy Website** for specific target audiences, namely pregnant individuals and their family.

During this interview, you will be asked to complete tasks read aloud to you by myself.

Think-Aloud Protocol

We will employ a task-based think-aloud protocol, in which we will ask you to communicate your thought processes verbally while you work. We will ask you to vocalize what path you take to find information, what questions you have, and what surprises or confuses you as you go through the website. We will keep questions open-ended and neutral, such as "What do you mean by that?" or "What did you expect to happen?" When you identify a problem, we will ask you how you would fix it. We will observe body language and facial expressions as well.

At this point I will ask you to share your screen with me and open the website.

<paste website address in the chat>

Possible Questions (may vary once website is fully developed)

<table>
<tr><th colspan="2">1. Finding Information about Influenza Vaccination in Pregnancy</th></tr>
<tr><td colspan="2">Scenario: You have recently found out that you are pregnant. You visited your family doctor and they recommended that you visit this website to learn more about influenza in pregnancy. Please use the website as you would to find more information about influenza in pregnancy.</td></tr>
<tr><th>Possible process and prompts</th><th>Notes</th></tr>
<tr><td>[Facilitator: Have "homepage" open. Remind the user we are testing the software, not them. Encourage them to think aloud. ("what words are going thru your mind?", "what are you looking for?")]</td><td></td></tr>
<tr><td>[Facilitator notes each step user clicks]</td><td></td></tr>
<tr><td>[Facilitator notes verbal comments:]</td><td></td></tr>
<tr><td>Difficulty rating of this task:
<table><tr><td>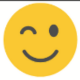</td><td>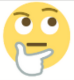</td><td>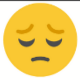</td><td>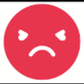</td></tr><tr><td>0</td><td>1</td><td>2</td><td>3</td></tr></table></td><td></td></tr>
</table>

0 = User completed task with zero difficulty. (Zero Frustration)<br>1 = User completed task with only minor problem(s). (Little Frustration)<br>2 = User completed task, but it required more effort/time/dead-ends than the user expected. (Medium/High Frustration)<br>3 = User did not complete task. (Point of Failure)	
Time completing this task: ______	
[**Facilitator** asks if any suggestions for making this (task) easier?]	
[**Facilitator asks –** Please take some time to read the content on *Influenza*, is it clear, understandable, respectful, does it leave you with more questions or are you satisfied with the level of detail]	

***2.* Finding Information about Vaccines in the Second Trimester**	
Scenario: *You are in your second trimester, up until now, your doctor has not spoken to you about vaccination. You find the Canadian Vaccination in Pregnancy Website and would like to learn more about which vaccines you should be considering in your second trimester. Please use the website as you would to find more information about second trimester vaccines in pregnancy.*	
**Possible process and prompts**	**Notes**
[**Facilitator:** Have "homepage" open. Remind the user we are testing the software, not them. Encourage them to think aloud. ("what words are going thru your mind?", "what are you looking for?")]	
[**Facilitator** notes each step user clicks]	
[**Facilitator** notes verbal comments:]	

Difficulty rating of this task:<br><table><tr><td>0</td><td>1</td><td>2</td><td>3</td></tr></table>0 = User completed task with zero difficulty. (Zero Frustration)<br>1 = User completed task with only minor problem(s). (Little Frustration)<br>2 = User completed task, but it required more effort/time/dead-ends than the user expected. (Medium/High Frustration)<br>3 = User did not complete task. (Point of Failure)	
Time completing this task: _______	
[**Facilitator** asks if any suggestions for making this (task) easier?]	
[**Facilitator asks –** Please take some time to read the content on *Second Trimester*, is it clear, understandable, respectful, does it leave you with more questions or are you satisfied with the level of detail]	

**3. Finding Information about Vaccines as a support person**	
Scenario: *You are a support person, you would like some tips on how to support your loved one in their vaccination in pregnancy decision. Please use the website as you would to find more information about tips for a support person in pregnancy.*	
**Possible process and prompts**	**Notes**
[**Facilitator:** Have "homepage" open. Remind the user we are testing the software, not them. Encourage them to think aloud. ("what words are going thru your mind?", "what are you looking for?")]	

<table>
<tr><td>[Facilitator notes each step user clicks]</td><td></td></tr>
<tr><td>[Facilitator notes verbal comments:]</td><td></td></tr>
<tr><td>Difficulty rating of this task:

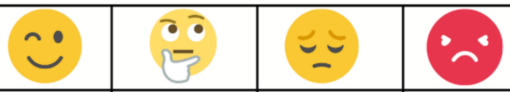
<table>
<tr><td>0</td><td>1</td><td>2</td><td>3</td></tr>
</table>
0 = User completed task with zero difficulty. (Zero Frustration)
1 = User completed task with only minor problem(s). (Little Frustration)
2 = User completed task, but it required more effort/time/dead-ends than the user expected. (Medium/High Frustration)
3 = User did not complete task. (Point of Failure)</td><td></td></tr>
<tr><td>Time completing this task: _______</td><td></td></tr>
<tr><td>[Facilitator asks if any suggestions for making this (task) easier?]</td><td></td></tr>
<tr><td>[Facilitator asks – Please take some time to read the content on Support Person, is it clear, understandable, respectful, does it leave you with more questions or are you satisfied with the level of detail]</td><td></td></tr>
</table>

<table>
<tr><th colspan="2">4. Finding Information about Covid-19 vaccines</th></tr>
<tr><td colspan="2">Scenario: You are in your first trimester and your partner is having some Covid-19 symptoms. You have been hesitant and are not vaccinated but now you feel concerned and would like to explore what are the options to protect yourself now that you are expecting a baby.</td></tr>
<tr><th>Possible process and prompts</th><th>Notes</th></tr>
<tr><td></td><td></td></tr>
</table>

[**Facilitator:** Have “homepage” open. Remind the user we are testing the software, not them. Encourage them to think aloud. (“what words are going thru your mind?”, “what are you looking for?”)]	
[**Facilitator** notes each step user clicks]	
[**Facilitator** notes verbal comments:]	
Difficulty rating of this task:<br>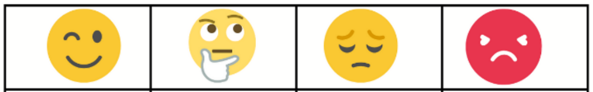<br>**0** | **1** | **2** | **3**<br>0 = User completed task with zero difficulty. (Zero Frustration)<br>1 = User completed task with only minor problem(s). (Little Frustration)<br>2 = User completed task, but it required more effort/time/dead-ends than the user expected. (Medium/High Frustration)<br>3 = User did not complete task. (Point of Failure)	
Time completing this task: _______	
[**Facilitator** asks if any suggestions for making this (task) easier?]	
[**Facilitator asks –** Please take some time to read the content on *Covid-19 vaccines*, is it clear, understandable, respectful, does it leave you with more questions or are you satisfied with the level of detail]	

**5. Finding Information mRNA Vaccines**

<table>
<tr><td colspan="2">Scenario: You have heard that mRNA vaccines use a new technology, and you are not sure if it is safe for you and your baby to have an mRNA vaccine. Please use the website as you would to find more information about how mRNA vaccines work and if they are safe in pregnancy.</td></tr>
<tr><th>Possible process and prompts</th><th>Notes</th></tr>
<tr><td>[Facilitator: Have “homepage” open. Remind the user we are testing the software, not them. Encourage them to think aloud. (“what words are going thru your mind?”, “what are you looking for?”)]</td><td></td></tr>
<tr><td>[Facilitator notes each step user clicks]</td><td></td></tr>
<tr><td>[Facilitator notes verbal comments:]</td><td></td></tr>
<tr><td>Difficulty rating of this task:
<table><tr><td>0</td><td>1</td><td>2</td><td>3</td></tr></table>0 = User completed task with zero difficulty. (Zero Frustration)
1 = User completed task with only minor problem(s). (Little Frustration)
2 = User completed task, but it required more effort/time/dead-ends than the user expected. (Medium/High Frustration)
3 = User did not complete task. (Point of Failure)</td><td></td></tr>
<tr><td>Time completing this task: _______</td><td></td></tr>
<tr><td>[Facilitator asks if any suggestions for making this (task) easier?]</td><td></td></tr>
<tr><td></td><td></td></tr>
</table>

[**Facilitator asks –** Please take some time to read the content on *Covid-19 vaccines*, is it clear, understandable, respectful, does it leave you with more questions or are you satisfied with the level of detail]	
