# Supplementary material for: Supporting Informed Vaccine Decision-Making and Communication in Pregnancy Through the Vaccines in Pregnancy Canada Intervention: Multimethod Co-Design Study
Source: J Med Internet Res. 2025 Dec 16;27:e77446. doi: 10.2196/77446 (PMC12754583; doi:10.2196/77446)
Supplement: Multimedia Appendix 5 [file jmir_v27i1e77446_app5.pdf]

UNIVERSITY OF CALGARY

UNIVERSITY OF CALGARY

CO-DESIGN PROVIDER COMPONENT

CONTENT AND USABILITY TESTING SURVEYS–VIP SKILLS COURSE (LMS)

PART 1: VIP Skills Course Online Modules Evaluation Questionnaire

Introduction

Thank you for completing this module. We would appreciate it if you could take a moment to provide your feedback.

Layout

Was the layout of this module easy to follow?

- ☐ Yes
- ☐ No

Information Quantity

Was the amount of information in this module adequate?

- ☐ Too Little
- ☐ Just Right
- ☐ Too Much

Readability

Was the text easy to read and understand?

- ☐ Yes
- ☐ No

Comprehensiveness

Did this module cover *the topic* comprehensively?

- ☐ Yes
- ☐ No

Inclusion

The language of this module is inclusive (gender, cultural background etc).

☐ Strongly Disagree
☐ Disagree
☐ Neutral
☐ Agree
☐ Strongly Agree

The images of this module are inclusive (gender, cultural background etc).

☐ Strongly Disagree
☐ Disagree
☐ Neutral
☐ Agree
☐ Strongly Agree

How can we make this module more inclusive and respectful?
Type text

Satisfaction

Overall, how satisfied are you with this module?

☐ Dissatisfied
☐ Neutral
☐ Satisfied

Additional Comments

Any additional comments or suggestions for improvement?

Type Text

PART 2: Overall Course Evaluation Questionnaire

Introduction

Thank you for taking the time to complete this questionnaire. Your feedback is valuable and will help improve this course. Please answer the following questions honestly, your responses will be kept anonymous.

Layout

How would you rate the overall layout of the *insert name* course?
☐ Very Poor
☐ Poor

- ☐ Neutral
- ☐ Good
- ☐ Excellent

How easy was it to navigate through the course?

- ☐ Very Difficult
- ☐ Difficult
- ☐ Neutral
- ☐ Easy
- ☐ Very Easy

The arrangement of menus and options was intuitive.

- ☐ Strongly Disagree
- ☐ Disagree
- ☐ Neutral
- ☐ Agree
- ☐ Strongly Agree

Amount of Information

The information in the course was overwhelming.

- ☐ Strongly Disagree
- ☐ Disagree
- ☐ Neutral
- ☐ Agree
- ☐ Strongly Agree

I found the amount of information available adequate for my learning needs.

- ☐ Strongly Disagree
- ☐ Disagree
- ☐ Neutral
- ☐ Agree
- ☐ Strongly Agree

Readability

How would you rate the readability of text and content?

- ☐ Very Poor
- ☐ Poor
- ☐ Neutral
- ☐ Good

- ☐ Excellent

Were there any sections where you found it difficult to understand the language used?

- ☐ Yes (Please specify: _________________)
- ☐ No

Comprehensiveness

The course covered all the topics I expected.

- ☐ Strongly Disagree
- ☐ Disagree
- ☐ Neutral
- ☐ Agree
- ☐ Strongly Agree

The content was in-depth enough to understand the subject matter.

- ☐ Strongly Disagree
- ☐ Disagree
- ☐ Neutral
- ☐ Agree
- ☐ Strongly Agree

Inclusion

The language of this course is inclusive (gender, cultural background etc).

- ☐ Strongly Disagree
- ☐ Disagree
- ☐ Neutral
- ☐ Agree
- ☐ Strongly Agree

The images of this course are inclusive (gender, cultural background etc).

- ☐ Strongly Disagree
- ☐ Disagree
- ☐ Neutral
- ☐ Agree
- ☐ Strongly Agree

How can we make this course more inclusive and respectful?
Type text

Perceived Satisfaction

Overall, how satisfied are you with the platform used to deliver the online components of the course?

- ☐ Very Dissatisfied
- ☐ Dissatisfied
- ☐ Neutral
- ☐ Satisfied
- ☐ Very Satisfied

What features did you particularly like?
Type text

What features do you think need improvement?
Type text

Additional Questions

Would you consider an in-person skills practice session (role-playing, case studies, debriefing) will enhance the objectives of this course?

- ☐ Definitely Not
- ☐ Probably Not
- ☐ Neutral
- ☐ Probably Yes
- ☐ Definitely Yes

As a course participant, would you be willing to participate in an in-person skills practice session?

- ☐ Definitely Not
- ☐ Probably Not
- ☐ Neutral
- ☐ Probably Yes
- ☐ Definitely Yes

Do you have any additional comments regarding the course?
Type text

Thank you for completing this questionnaire. Your feedback is valuable to us!
