# Supplementary material for: Supporting Informed Vaccine Decision-Making and Communication in Pregnancy Through the Vaccines in Pregnancy Canada Intervention: Multimethod Co-Design Study
Source: J Med Internet Res. 2025 Dec 16;27:e77446. doi: 10.2196/77446 (PMC12754583; doi:10.2196/77446)
Supplement: Multimedia Appendix 6 [file jmir_v27i1e77446_app6.pdf]

VIP CANADA INTERVENTION COMPONENTS AND BEHAVIORAL TARGETS AND TECHNIQUES

Component description	Behavioral change techniques used	Target domains
**DECIDE COMMUNICATION APPROACH**		
• DECIDE acronym outlining clear steps and instructions on how to approach vaccine communications<br>• Tips and conversation starters.	Instructions on how to perform behavior	Skills<br>Knowledge
**VIP SKILLS COURSE FOR PROVIDERS**		
Introductory group session		
Expert video – Introduction<br>• The video is introduced by an expert who is considered a credible source.<br>• The video explains the relevance of vaccination during pregnancy<br>• Reinforce the importance of providers' role in vaccination.	Verbal persuasion about capability<br><br>Credible source<br><br>Information about antecedents<br><br>Identification of self as a role model<br><br>Information about social consequences<br><br>Info about health consequences	Social influences<br><br>Knowledge<br><br>Beliefs about capabilities<br><br>Social professional role and identity<br><br>Beliefs about consequences
Self-reflection<br>• Providers will be asked to reflect on their vaccine communications, if they want to improve how they think they can improve, and when and where they consider that can	Action planning<br><br>Problem solving	Beliefs about capabilities<br><br>Goals

introduce vaccine communications to their practices.		
Facilitated discussion and reflection.<br>• The clinic champion (who was previously trained in component # 3) will present the slide deck that includes more information about vaccination in pregnancy.<br>• Includes a reflection and discussion about previous experiences of vaccination in pregnancy communications and witnessing the effects of non-vaccination during pregnancy. The challenges that participants mention will be used to reflect on why improving vaccine communications is needed.<br>• This includes a discussion of how vaccines are currently used in pregnancy communications (this will help to inform the environmental scan and the mapping).<br>• A slide about the skills that providers need to be successful in vaccine communications will be presented priming them for success by pointing out that they already have what is needed and the course will strenghten this.<br>• A video testimonial from a patient will be presented to emphasize why improving is necessary from the perspective of a patient with lived experience.	Information about antecedents.<br><br>Information about health consequences.<br><br>Focus on past success.<br><br>Salience of consequences<br><br>Information about social and environmental consequences<br><br>Problem solving<br><br>Credible source	Knowledge<br><br>Beliefs about capabilities<br><br>Social influences<br><br>Beliefs about consequences<br><br>Reinforcement

Module 1: Why vaccinate in pregnancy?		
• The content is focused on why to vaccinate during pregnancy (Increased morbidity and mortality, physiology, and mechanisms of action, etc.)<br>• Includes readings to support the provided information.<br>• Finalize with a call-to-action inviting providers to have vaccination in pregnancy communications.	Information about antecedents<br><br>Information about health consequences.<br><br>Credible source<br><br>Goal setting	Knowledge<br><br>Beliefs about consequences<br><br>Social professional role and identity<br><br>Social influences<br><br>Goals
Module 2: Introduction to DECIDE		
• Share research findings about the unique experience of decision-making in pregnancy including a video testimonial from a patient.<br>• Includes a self-reflection about why providers are taking the course and what they hope to get from it.<br>• Includes a video presenting two possible scenarios and the impact of them on vaccine decision-making, highlighting the importance of having vaccine communications early and often during pregnancy.<br>• Presents an overview of vaccine communication strategies (presumptive and participatory), showing findings about how parents living in Canada would prefer.	Information about antecedents.<br><br>Information about health consequences.<br><br>Goal setting<br><br>Salience of consequences<br><br>Information about social and environmental consequences<br><br>Demonstration of behavior.<br><br>Comparative imagining of future outcomes.<br><br>Credible source	Knowledge<br><br>Goals<br><br>Beliefs about capabilities<br><br>Social influences<br><br>Beliefs about consequences<br><br>Social professional role and identity.

• Highlights the need for a balanced communication strategy tailored for vaccines in pregnancy.<br>• Introduce DECIDE, a novel evidence-informed communication approach specific to vaccine decision-making during pregnancy.		
Module 3: Steps of the DECIDE communication approach.		
• Presents a structured breakdown of each DECIDE step.<br>• Includes a demonstration video for each step.<br>• Provides practical tools including tips and conversation starters for each step.<br>• Include multiple choice questions to verify if learners and understand the content and provide immediate feedback on their answers.	Information about antecedents.<br><br>Demonstration of behavior.<br><br>Instructions on how to perform the behavior.<br><br>Mental rehearsal of successful performance OR problem solving<br><br>Feedback on behavior	Knowledge<br><br>Skills<br><br>Social professional role and identity<br><br>Beliefs about consequences<br><br>Beliefs about capabilities
Module 4: Practice scenarios		
• Includes practice scenarios for providers to rehearse the DECIDE communication approach.<br>• Feedback will be provided for providers to compare their answers.<br>• It contains a self-reflection piece about participants' performance of the simulation games and the impact that this can have on their clinical practice.	Self-monitoring of behavior<br><br>Behavioral practice<br><br>Feedback on behavior<br><br>Comparative imagining of future outcomes<br><br>Problem-solving	Knowledge<br><br>Beliefs about consequences<br><br>Beliefs about capabilities<br><br>Social professional role and identity<br><br>Skills
Final practice group session		

Debrief from modules and DECIDE recap<br>• Discussion around main learnings from the self-directed learning modules.<br>• The clinic champion will present a slide deck with a recap of the DECIDE approach including clear instructions for each step.	Instructions on how to perform a behavior.	Knowledge
Practice with peers<br>• A case study will be presented to discuss how to approach that specific vaccination conversation.<br>• Providers will participate in a role-playing activity to practice the DECIDE approach in different scenarios followed by a debrief where they will provide mutual feedback.<br>• Providers complete a self-assessment form of their performance in the activity.	Behavioral practice<br><br>Feedback on behavior<br><br>Self-monitoring of behavior<br><br>Problem solving	Skills<br><br>Knowledge<br><br>Beliefs about consequences<br><br>Beliefs about capabilities<br><br>Social professional role and identity
Professional credits and certificate<br>• If providers meet all the requirements, after the second in person session they will get professional credits and certificate for the completion of the training.	Non-specific reward	Social professional role and identity<br><br>Reinforcement
Vaccination in Pregnancy Champion Badge<br>• At the end of the in-person session, providers will receive a badge as a "Vaccination in Pregnancy Champion". This object will act as a reminder of their role and the skills they have to have a	Prompts # cues<br><br>Adding objects to the environment	Social influences<br><br>Social professional role and identity.

vaccination in pregnancy communications.		
**PRACTICE CHANGE PLAN**		
Initial engagement<br>• General presentation of the intervention (background including relevance of vaccination during pregnancy, procedures, and expected outcomes and benefits of using the intervention)<br>• Identification of a Clinic Champion to support the intervention: A local champion will facilitate the intervention delivery at the local site; ideally a person within the clinic who staff trust and value as a team member	Credible Source	Social influences
Operational champion engagement throughout the intervention<br><br>• Orientation with the clinic champion to explain including an explanation of their role in the intervention.<br>• First and second synchronous session preparation: Engage the champion by explaining what is going to happen in the synchronous session, schedule it and determine mode of delivery preferred by the clinic (in person, virtual).<br>• First and second debriefing session: to get insights and feedback about the synchronous session and explain next steps.	Identification of self as a role model<br><br>Credible source	Social influences<br><br>Social professional role and identity

• The champion will be engaged on the review of current state.		
Review of current state of vaccine in pregnancy communications.<br>• Mapping to capture current patient flow and characterize how are vaccine in pregnancy communications happening (who is initiating the conversation, when and where is happening, existing materials).<br>• Discuss readiness to change the current procedures with the goal of making vaccine communications part of routine prenatal care.	Action planning<br><br>Commitment	Environmental Context and Resources<br><br>Intentions
Consensus session<br>• Providers will generate a plan to integrate vaccine communication into practice.<br>• The plan is created through consensus and will determine process changes (how vaccine in pregnancy communications are going to happen: who is initiating the conversations, when and where will happen) and environment modifications needed (which materials are needed in the physical space to support the plan)<br>• Providers will make the commitment to follow the plan.	Goal setting (behavior)<br><br>Goal setting (outcome)<br><br>Action planning<br><br>Commitment	Environmental Context and Resources<br><br>Intentions<br><br>Goals<br><br>Social professional role and identity
Environmental modification	Adding objects to the environment	Environmental Context and Resources

• The practice change plan will be printed and placed in a place where is visible to providers.<br>• Printed materials will be accessible in the clinic and placed in the more appropriate spots according to the practice change plan.	Instructions on how to perform a behavior.<br><br>Restructuring the physical environment<br><br>Restructuring the social environment.	Memory, attention and decision processes<br><br>Goals<br><br>Intentions
VIP CANADA WEBSITE		
FAQ, vaccine-specific content, and trimester-specific sections.<br><br>• Contains information about vaccine mechanisms of action, components, safety, and myths vs facts.<br>• Includes information about the importance of vaccination during pregnancy.<br>• Specify information about what to do if people decide to get the vaccines (timing for the vaccines, where to get the vaccines, and what to expect)<br>• Includes quotes from patients allowing pregnant people to value peers experiences.	Information about health consequences<br><br>Instructions on how to perform a behavior.<br><br>Information about antecedents<br><br>Social comparison	Knowledge<br><br>Social influences
Video testimonials<br><br>• We included patient testimonials because of the value and credibility that peers' experiences have for pregnant people.<br>• The testimonials include situations that pregnant people can relate to.	Credible source<br><br>Social comparison	Social Influences

Infographics<br><br>• Contains information about vaccine mechanisms of action, components, safety, and myths vs facts.<br>• Includes information about the importance of vaccination during pregnancy.<br>• Specify information about what to do if people decide to get the vaccines (timing for the vaccines)<br>• It includes the logos of the organizations that supports the project as a way to endorse and give credibility to the website.	Information about health consequences<br><br>Instructions on how to perform a behavior.<br><br>Credible source	Knowledge<br>Social influences
How vaccines work video<br>• The video is introduced by an expert who is considered a credible source.<br>• Include a plain-language explanation of the vaccines' mechanism of action to highlight how vaccines protect the pregnant person and their baby.<br>• Emphasizes the fact that vaccine components don’t reach the baby and are eliminated by the pregnant person's organism while mom and baby get all the benefits from the antibodies.	Information about antecedents<br><br>Credible source	Knowledge<br>Social influences
References and logos	Credible source	Social influences

• It includes references to support that the website content is evidence-based.<br>• The logos from the organizations and institutions that support the website are included at the bottom of the website as an endorsement of the content.		
Support person section<br><br>• This content presents suggestions about what actions a support person can take to support a pregnant person (e.g. get their own vaccines).<br>• Provide information about the importance of the support person role and how this benefits the pregnant person.	Instructions on how to perform the behavior.<br><br>Valued self-identity<br><br>Verbal persuasion about capabilities<br><br>Information about emotional consequences<br><br>Instructions on how to perform the behavior.<br><br>Information about health consequences<br><br>Information about social consequences	Social Influences<br>Knowledge,<br>Social professional role and identity<br>Beliefs about capabilities<br>Beliefs about consequences
Pictures and graphics<br>• The pictures and graphics show diversity and inclusivity by incorporating persons from different genders and cultural backgrounds.<br>• Website users will identify and relate to the images as a way to point out that the website is for people "like them".	Restructuring social environment<br><br>Social comparison	Social influences<br>Social professional role and identity
