# Supplementary material for: Supporting Informed Vaccine Decision-Making and Communication in Pregnancy Through the Vaccines in Pregnancy Canada Intervention: Multimethod Co-Design Study
Source: J Med Internet Res. 2025 Dec 16;27:e77446. doi: 10.2196/77446 (PMC12754583; doi:10.2196/77446)
Supplement: Multimedia Appendix 7 [file jmir_v27i1e77446_app7.pdf]

VACCINES IN PREGNANCY CANADA
Protection for you is protection for two+

Skills Practice Session
Debriefing tool

Scenario # __

Adapted from: *David Crookall. Debriefing: A practical guide. Simulation for Participatory Education: Virtual Exchange and Worldwide Collaboration, Springer, 2023, Springer Texts in Education, 978-3-031-21010-5. ⟨10.1007/978-3-031-21011-2_6⟩. ⟨hal-03904518v1⟩*

Observer guide for PROFFIteROLE[1]	Role	First Name
Remember that this guide is not a strict evaluation instrument. It is a guide to help observers organize their observations. Interpretation of the terms in this guide and the observations remain subjective. The guide collects impressions to serve as a starting point for discussion in the debriefing.<br><br>Remember it is not necessary for the HCP to address each tip in the box below. Please assess as you see appropriate.	During each scenario, you will rotate through each role:<br><br>***Circle One****<br><br>Health Care Provider<br><br>Patient<br><br>Observer	

	Good	Fair	Improve
**DETERMINE** if your patient is aware and ready to talk about vaccination.<br><br>*Things to think about:*<br><br>Did the HCP:<br>• Reinforce vaccination is a routine part of perinatal care.<br>• Introduce vaccination by connecting to other preventative perinatal interventions i.e. prenatal vitamins.			
• Acknowledge that it is normal to have questions.			
**ELICIT** your patient's questions.<br><br>*Things to think about:*<br><br>Did the HCP:<br>• Show that they were actively listening or affirming their patient's perspective.<br>• Validating feelings, even if they disagreed with their patient's opinion.<br>• Asked probing questions to understand perspective.			
**CONSENT** to share information & initiate vaccine conversation.<br><br>*Things to think about:*<br><br>Did the HCP:<br>• Normalize and validate concern.<br>• Tailor their consent statement<br>• Reaffirm their common goal: to keep both their patient and baby healthy.			
**INTERACTIVE DISCUSSION** to address your patient's specific questions.<br><br>*Things to think about:*<br><br>Did the HCP use any of the following messages, as appropriate:<br>• "Protection for you is protection for two."<br>• "We don't know everything, but we know enough."			
• "The vaccine components don't get to baby."<br>• "Vaccines build on what our bodies do naturally.<br><br>Did the HCP:<br>• Address their patient's questions.<br>• Tailor their response to the patient's needs<br>• Combine strategies to address multiple "C's"			
**DELIVER** recommendation that considers. your patient's perspectives.<br><br>*Things to think about:*<br><br>Did the HCP:<br>• Emphasize: "Protection for you is protection for two."<br>• Explain that other reputable groups, like the Society of Obstetricians and Gynaecologists of Canada (SOGC) also recommend vaccination during pregnancy.<br>• Deliver your vaccine recommendation, affirm your patient's autonomy, then restate your recommendation and offer ongoing support.			
**EMPOWER** patient to take next step towards an informed decision.<br><br>*Things to think about:*			
Did the HCP:<br>• Suggest taking time to reflect and weigh available options.<br>• Offer a list of trustworthy resources via a pamphlet or fact sheet.<br>• Offer to book a follow-up appointment to address additional questions; wrap-around support			
Welcome, attitude, approach, friendliness			
Additional Comments or Feedback			

Reflective Questions

Read the questions on your own <u>after</u> the simulation learning has taken place. Write your responses to the following questions:

1. Describe the knowledge or skills that you felt were consistent with the current evidence.

2. Describe opportunities for improvement that you have identified during the simulation.

3. What learning strategies will you pursue to address the areas for improvement?

4. <u>Describe an action plan*</u> to implement proposed improvements, including any anticipated barriers to change.
