# Supplementary material for: Supporting Informed Vaccine Decision-Making and Communication in Pregnancy Through the Vaccines in Pregnancy Canada Intervention: Multimethod Co-Design Study
Source: J Med Internet Res. 2025 Dec 16;27:e77446. doi: 10.2196/77446 (PMC12754583; doi:10.2196/77446)
Supplement: Multimedia Appendix 8 [file jmir_v27i1e77446_app8.pdf]

Practice change plan template

VACCINES IN PREGNANCY CANADA

Action plan for your clinic

**WHEN** Indicate when this action should take place (e.g., at the first appointment, when a vaccine is due, during a specific trimester or visit).	**WHO** Specify the person or group in charge of this task)	**HOW** Describe the follow-up action here (e.g., people will receive a vaccine reminder).	**MATERIALS NEEDED** List the decision aids needed to support this step (e.g., poster, pamphlet, QR code, video)	**REMINDERS** Explain how people will be reminded to complete the action (e.g., checkmark, system notification, etc.).
				.

VACCINES IN PREGNANCY CANADA

Environment modification

In this step, consider two key elements: (1) the physical spaces that can be adapted to support the action plan, and (2) the decision aids available. Then, decide which decision aids should be placed in each space to create an environment that supports the action.
(Example: you might place an infographic in the waiting room, a poster in the washroom, or a pamphlet at the reception desk)

Waiting Room	Reception	Washroom
**Examination Room**	**Doctors and nurse's station**	**Other places**

VACCINES IN PREGNANCY CANADA
